# Investigating time-*independent* and time-*dependent* diffusion phenomena using steady-state diffusion MRI

**DOI:** 10.1038/s41598-025-87377-x

**Published:** 2025-01-28

**Authors:** Benjamin C. Tendler

**Affiliations:** Wellcome Centre for Integrative Neuroimaging, FMRIB, Nuffield Department of Clinical Neurosciences, University of Oxford, Oxford, UK

## Abstract

Diffusion MRI is a leading method to non-invasively characterise brain tissue microstructure across multiple domains and scales. Diffusion-weighted steady-state free precession (DW-SSFP) is an established imaging sequence for post-mortem MRI, addressing the challenging imaging environment of fixed tissue with short T_2_ and low diffusivities. However, a current limitation of DW-SSFP is signal interpretation: it is not clear what diffusion ‘regime’ the sequence probes and therefore its potential to characterise tissue microstructure. Building on Extended Phase Graphs (EPG), I establish two alternative representations of the DW-SSFP signal in terms of (1) conventional b-values (time-*independent* diffusion) and (2) encoding power-spectra (time-*dependent* diffusion). The proposed representations provide insights into how different parameter regimes and gradient waveforms impact the diffusion sensitivity of DW-SSFP. I subsequently introduce an approach to incorporate existing biophysical models into DW-SSFP without the requirement of extensive derivations, with time dependence estimated via a Gaussian phase approximation representation of the DW-SSFP signal. Investigations incorporating free-diffusion and tissue-relevant microscopic restrictions (cylinder of varying radius) give excellent agreement to complementary analytical models and Monte Carlo simulations. Experimentally, the time-*independent* representation is used to derive Tensor and proof-of-principle NODDI estimates in a whole human post-mortem brain. A final SNR-efficiency investigation demonstrates the theoretical potential of DW-SSFP for ultra-high field microstructural imaging.

## Introduction

**Fig. 1:**
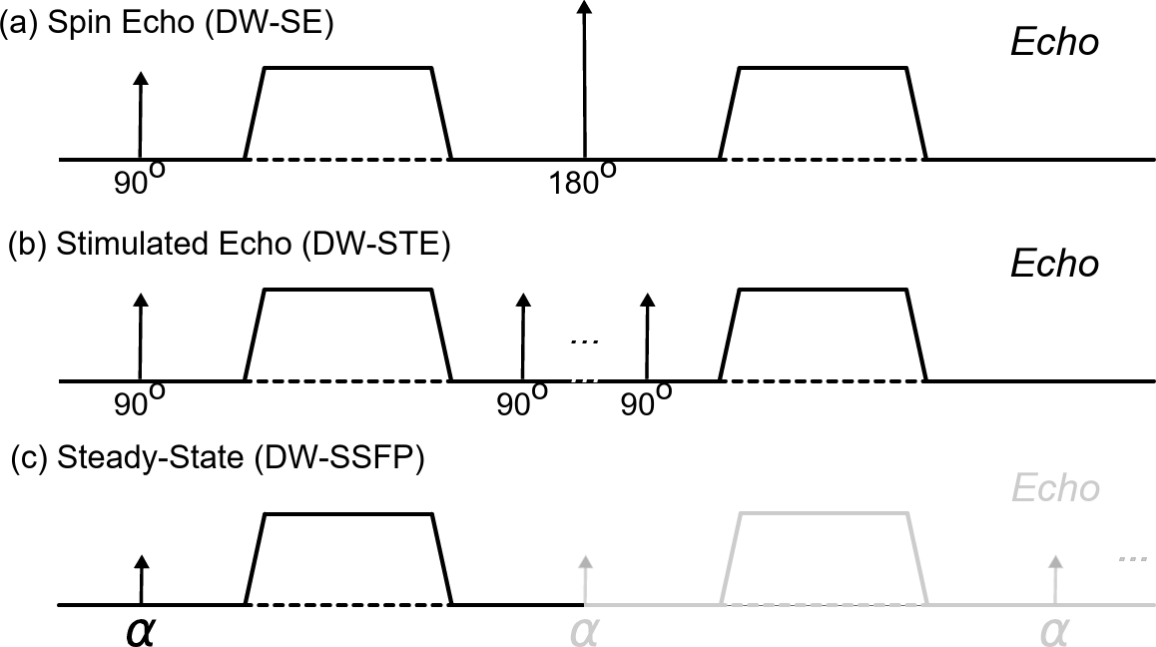
Diffusion encoding of different MRI sequences. The diffusion-weighted spin-echo (DW-SE) (**a**); diffusion-weighted stimulated echo (DW-STE) (**b**); and diffusion-weighted steady-state free precession (DW-SSFP) (**c**) sequence. DW-STE (**b**) achieves strong diffusion-weighting with reduced T_2_ signal loss by increasing diffusion sensitisation longitudinally (associated with slow T_1_ recovery). This results in increased experimental time and signal-forming mechanisms that lead to a twofold reduction in signal levels when compared to a DW-SE. (**c**) DW-SSFP consists of a single RF pulse and diffusion gradient per TR. Magnetisation persists and evolves over multiple TRs (see “Theory”), which can lead to high SNR-efficiency and strong diffusion weighting.

Establishing non-invasive neuroimaging technologies to characterise tissue microstructure facilitates investigations into the human brain and the efficacy of novel neurotherapeutic targets. Diffusion MRI is a leading neuroimaging modality in this space, probing tissue microstructure by sensitising images to changes in cellular morphology. At present, diffusion MRI investigations are almost exclusively performed using the diffusion-weighted spin-echo (DW-SE) MRI sequence^1^ (Fig. 1a). DW-SE provides an intuitive framework to understand and investigate diffusion across multiple domains and scales. More advanced diffusion acquisition schemes^2^ are often fundamentally based on the DW-SE, making it the principal technique to non-invasively characterise tissue microstructure.

While DW-SE is the leading diffusion MRI method, the sequence has several well-established limitations. These limitations often arise from its (1) long diffusion-encoding period and (2) EPI readout^2^. In recent decades, a key focus of MRI methods development has been the establishment of new technologies to address these limitations. This includes the development of (1) specialised gradient systems^3–5^, (2) accelerated acquisition methods^6^ and (3) novel image-reconstruction algorithms^6^.

Broadly, any MRI sequence can be made sensitive to diffusion with the correct placement of encoding gradients. Different sequences have their own advantages and disadvantages, and some offer potential benefits over conventional DW-SE that warrant exploration, particularly in domains where DW-SE struggles to achieve high diffusion-sensitivity or adequate SNR. One alternative is the diffusion-weighted stimulated-echo^7^ (DW-STE) sequence (Fig. 1b), achieving higher b-values with a typical trade-off of reduced SNR-efficiency, due primarily to a fundamental factor of two signal reduction compared to DW-SE.

In this work, I investigate Diffusion-Weighted Steady-State Free Precession^8–11^ (DW-SSFP) (Fig. 1c), a powerful diffusion imaging sequence that has demonstrated high SNR-efficiency and strong diffusion-weighting^12^ with minimal image distortions (no EPI readout required). Whilst in vivo use is plagued by high motion sensitivity^13^, DW-SSFP has become an established method for post-mortem imaging^14–22^, addressing the low-diffusivity and short T_2_ environment of fixed post-mortem tissue.

A key challenge for DW-SSFP is signal interpretation. Unlike conventional diffusion MRI sequences, DW-SSFP does not have a well-defined b-value^13,23,24^, and complicated dependencies on both tissue relaxation properties (T_1_ and T_2_) and sequence parameters (flip angle and TR)^13,25^. This means that DW-SSFP does not probe a well-defined diffusion ‘regime’ in as straightforward a manner as DW-SE, limiting both interpretation and the integration of biophysical models for characterising tissue microstructure. The deviation of DW-SSFP contrast from familiar DW-SE contrast mechanisms is embodied in the deviation of the diffusion-encoding module from conventional diffusion gradient pairs: DW-SSFP contains only a single diffusion gradient in each TR (Fig. 1c—black line).

In this paper, I first address the challenge of DW-SSFP signal interpretation. Building on Extended Phase Graphs (EPG)^26^, I establish two new representations of the DW-SSFP signal in terms of (1) conventional b-values (time-*independent* diffusion) and (2) encoding power-spectra^27^ (time-*dependent* diffusion). These representations build on existing DW-SSFP descriptions by incorporating the specific gradient waveform and timings experienced by distinct signal-forming pathways, facilitating translation of the DW-SSFP signal into an interpretable measurement (extending existing partition framework representations of DW-SSFP^11,25^). I use these representations to visualise how the DW-SSFP signal is impacted by both sequence parameters and alternative gradient waveforms (oscillating gradients), facilitating the identification of imaging regimes where the DW-SSFP signal has maximum sensitivity to tissue microstructure.

I subsequently use these representations to establish a framework for characterising the impact of time-*independent* and -*dependent* diffusion on the measured DW-SSFP signal. Here, time-*dependent* diffusion systems are characterised as a function of an arbitrary time-dependent diffusion coefficient ($D(t)$)^28^, investigated via a Gaussian phase approximation^27,29,30^ (GPA) representation of the DW-SSFP signal. Notably, existing biophysical models can be incorporated into the proposed framework without the requirement of extensive derivations. Investigations are performed considering both free Gaussian diffusion and a restriction system (cylinder of varying radius), achieving excellent agreement to existing analytical models of the DW-SSFP signal and Monte Carlo simulations.

From the perspective of signal-forming mechanisms, the proposed framework provides intuition into how DW-SSFP conceptually achieves higher levels of diffusion attenuation compared to a conventional DW-SE under the condition of matched diffusion encoding waveforms and timings. I apply the time-*independent* framework to experimental DW-SSFP data acquired in a whole post-mortem brain from a previously-described dataset^31,32^, estimating voxelwise tensor and proof-of-principle NODDI estimates.

Finally, I perform an investigation into the SNR-efficiency properties of DW-SSFP as a function of gradient strength, relaxation times, and encoding period (oscillating gradients). I find that whilst the theoretical SNR benefits of DW-SSFP are reduced when compared to DW-SE at increased gradient strengths (and matched encoding periods), conventional (unbalanced) DW-SSFP offers improved SNR-efficiency at short T_2_ and long T_1_ relaxation times. Combined with its low distortion properties, DW-SSFP demonstrates considerable promise for post-mortem microstructural imaging at ultra-high field.

Software is provided to enable researchers to perform their own investigations with the proposed frameworks, including scripts to replicate many of the findings presented in this manuscript (available at github.com/BenjaminTendler/SteadyStateDiffusionMicrostructure).

## Theory

### Overview of DW-SSFP

The DW-SSFP sequence consists of a single RF pulse (of flip angle $\alpha$) and diffusion gradient (with characteristic length scale ${q}^{-1}$) in each repetition time (Fig. 1c—black line)^13^. As with all steady-state sequences, the repetition times are short relative to the tissue T_2_ (TR typically 20–40 ms). Remaining transverse magnetisation is not spoiled at the end of each TR, leading to magnetisation that experiences repeated sensitisation to RF pulses and diffusion gradients (Fig. 1c—grey line).

In DW-SSFP, magnetisation accumulates diffusion contrast over several TRs, consistent with dephasing and rephasing of magnetisation due to gradient pairs in conventional diffusion MRI sequences. The resulting steady-state can be considered a superposition of multiple magnetisation components with different histories—e.g., pairs of diffusion-weighted gradients separated by a variable integer number of TRs that lead to different degrees of diffusion weighting. This creates a composite signal corresponding to a summation of these components, with non-trivial diffusion-weighted signal attenuation.

**Fig. 2:**
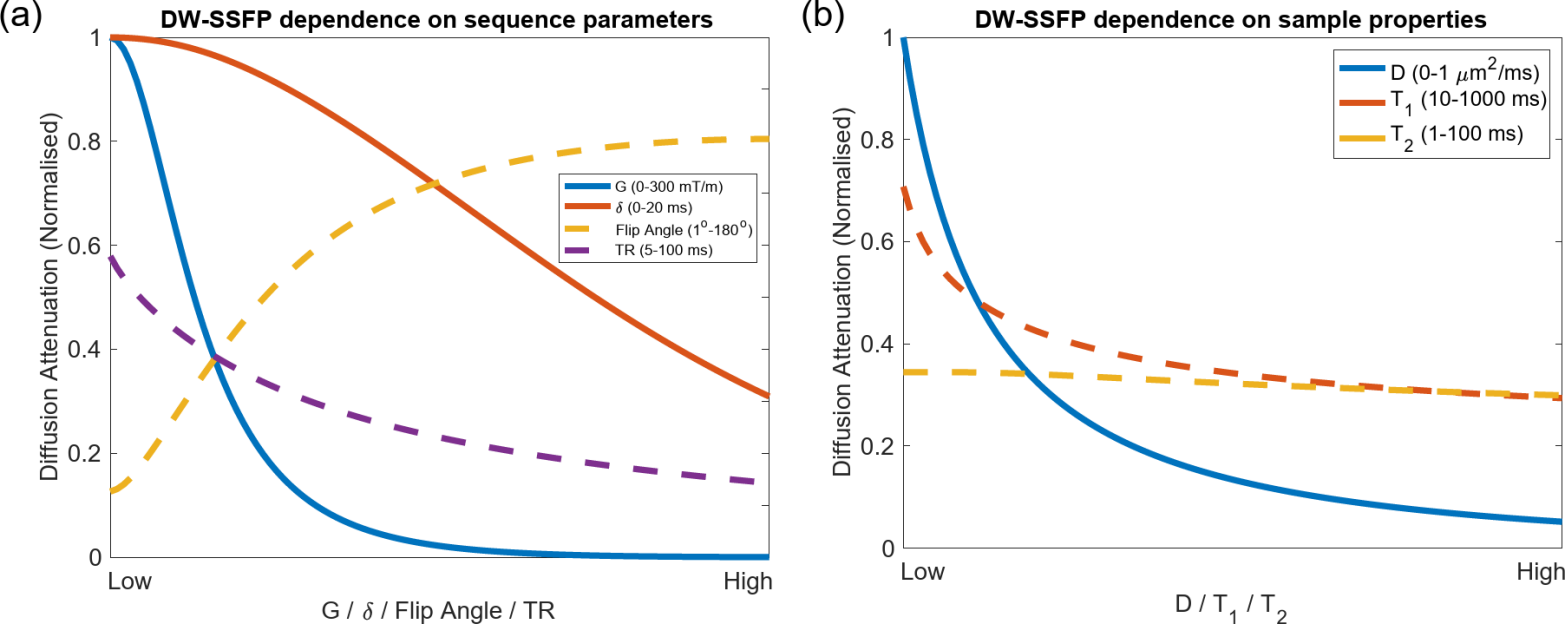
DW-SSFP diffusion attenuation. Dependence of DW-SSFP diffusion attenuation on (**a**) sequence parameters and (**b**) sample properties. Whilst conventional diffusion acquisitions are influenced by the diffusion gradient ($G$), duration ($\delta$) and coefficient ($D$) (solid lines), several further parameters influence DW-SSFP diffusion attenuation (dashed lines). In DW-SSFP, decreasing the flip angle/increasing the TR corresponds to increased diffusion-weighting (**a**). A higher T_1_ or T_2_ also leads to increased diffusion-weighting (**b**). DW-SSFP signal estimated using the analytical model described in Freed et al.^33^ with gradient duration $\delta$ (Appendix 1). Sequence parameters based on post-mortem DW-SSFP investigations at 7T as described in Tendler et al.^32^ (defined in “Methods”). Note that there is no explicit definition of diffusion time (${\Delta }$) for the DW-SSFP sequence. To explore these relationships further, find associated code here.

For unrestricted ‘free’ Gaussian diffusion, an analytical expression of the DW-SSFP signal exists^33^ (Appendix 1), analogous to, but far more complicated than, the characteristic exponential decay (${{S}_{0}e}^{-b\cdot D}$) associated with conventional diffusion MRI sequences. Examination of Appendix 1 provides insights into the dependencies of DW-SSFP. Diffusion-weighted terms (${e}^{-\dots D}$) are not separable from the remainder of the equation, leading to diffusion-attenuation additionally dependent on relaxation properties (T_1_ and T_2_), sequence flip angle (*α*), and TR (Fig. 2).

The DW-SSFP sequence can also be investigated using Extended Phase Graphs (EPG)^26^. Briefly, EPG describes a widely adopted framework for modelling the magnetisation evolution of an MRI sequence. It provides a solution to the Bloch equations^34^, reparameterising magnetisation as a Fourier basis with distinct phase states ($k$), defining:

- Longitudinal ($\widetilde{Z}$) and transverse ($\widetilde{F}$) magnetisation components with different phase ($k$) states.
- RF pulses acting as a mixing operator on ${\widetilde{Z}}_{k}$ and ${\widetilde{F}}_{k}$ components.
- Magnetic field gradients acting on ${\widetilde{F}}_{k}$ components.

EPG can characterise the estimated diffusion attenuation for an arbitrary train of RF pulses and diffusion gradients, providing a solution to the Bloch-Torrey equation^35^, with existing work incorporating both free Gaussian diffusion and extensions to diffusion anisotropy^26,36^. For readers unfamiliar with EPG signal representations, I recommend the review article by Weigel^26^.

### Characterising tissue microstructure with DW-SSFP

It is well established that diffusion in tissue is not in general characterised by free Gaussian diffusion^37^. Several features of tissue microstructure hinder and restrict the diffusion of water, the majority reflecting the presence of membranes with very limited permeability on the timescales of diffusion MRI measurements. This has motivated the development of sophisticated models, representations, and experimental methods to relate diffusion MRI measurements to microstructural features^37^.

In this work, I separate diffusion processes into two distinct domains. Time-*independent* diffusion processes characterise diffusion as Gaussian, where a sequence’s b-value is sufficient to fully describe the measured diffusion attenuation. This always holds for free-diffusion, and is also valid for microstructural systems in the long-diffusion time regime^37^. Time-*dependent* diffusion systems incorporate non-Gaussian diffusion processes (e.g. arising from restrictions experienced by mobile spins). Here, information about the specific gradient waveform timings, $G(t)$, are required to characterise the measured diffusion attenuation^37–39^.

For time-*independent* diffusion, previous DW-SSFP investigations have taken existing analytical representations of free Gaussian diffusion^25,33^ and extended them to multiple Gaussian compartments incorporating (1) tensors^14^, (2) ball and sticks^14,40^, and (3) a Gamma distribution of diffusivities^24^. Resulting estimates have been used to perform tractography^14,15,18,19,32^ and characterise the non-Gaussianity of tissue^24^. Previous investigations have utilised an approximation regime (two-transverse period approximation) of the DW-SSFP signal to demonstrate sensitivity to time-*dependent* restrictions^23,24^.

### Proposed DW-SSFP framework

In this manuscript I propose a framework that builds on EPG to characterise diffusion phenomena in DW-SSFP. This raises a key question to the motivation and potential benefits of introducing a new framework. Specifically, if the DW-SSFP signal can already be modelled using EPG, including extensions to diffusion anisotropy^26,36^, what does the new framework to address?

The new framework aims to address a central tenet of EPG, i.e. that signal-components with identical phase (*k*) states can be combined. Importantly, when considering diffusion, the summing of signal-components with identical phase states is only valid for systems characterised by free Gaussian diffusion (i.e. ${e}^{-bD}$). Only under these circumstances can the cumulative effect of diffusion sensitisation over several TRs be estimated via the matrix multiplication of the EPG diffusion operator (i.e. the product of ${e}^{-bD}$ terms).

**Fig. 3:**
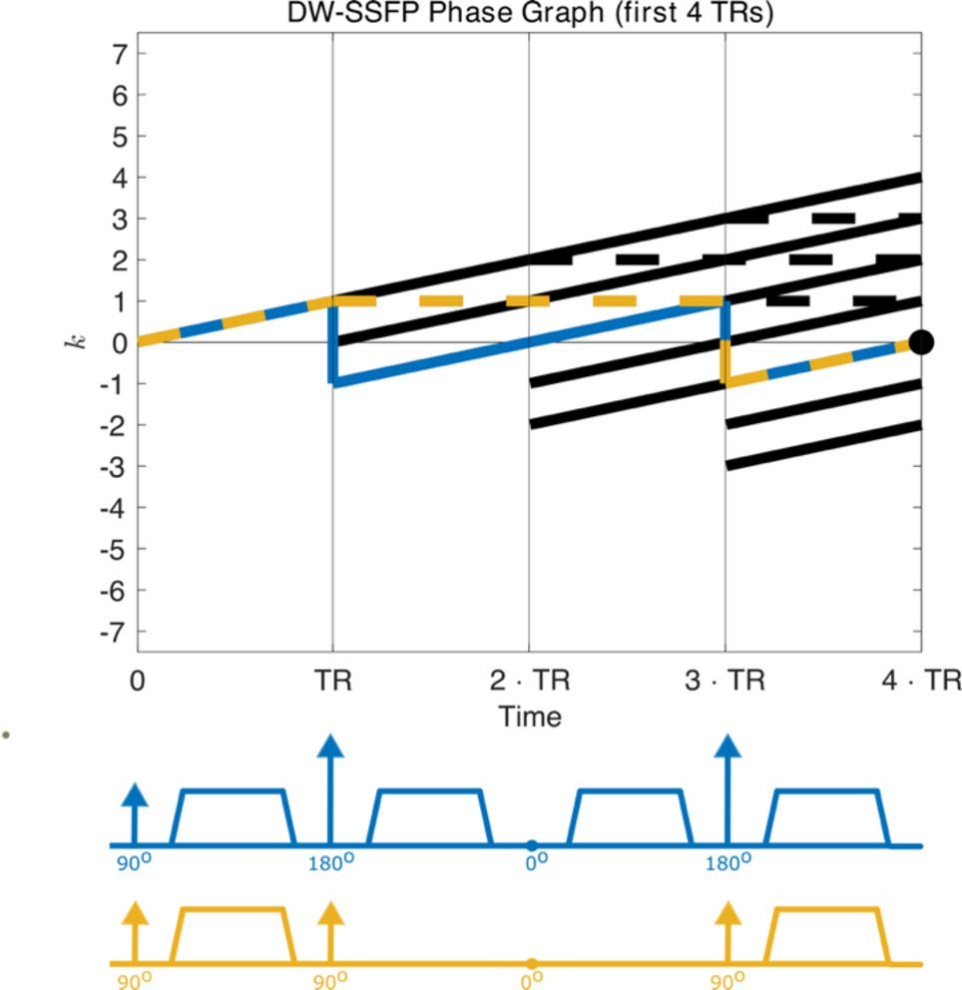
EPG representation of the DW-SSFP sequence. Using a phase graph representation^26^ of DW-SSFP (first four TRs), we can identify several pathways that contribute to the measured signal at the 4th TR. The two highlighted pathways (blue and yellow) have an identical phase state at the 4th TR (black dot), but distinct diffusion encoding trajectories (corresponding to double diffusion encoding^39^ and a stimulated echo^7^ respectively). Beyond free Gaussian diffusion, the b-value associated with the gradient waveform of these individual magnetisation pathways must be accounted for to accurately estimate the DW-SSFP signal arising from a time-*independent* system. The specific timings of the gradient waveform of these individual magnetisation pathways must be accounted for to accurately estimate the DW-SSFP signal arising from a time-*dependent* system, noting that even pathways with identical b-values but different gradient trajectories can lead to different signal estimates when considering time-*dependent* diffusion phenomena. This information is not preserved for individual magnetisation pathways within existing EPG frameworks, which combines magnetisation pathways with identical phase states and does not consider their evolution history.

This property arises as conventional EPG does not preserve information about the gradient waveform experienced by individual magnetisation pathways across multiple TRs. Specifically, in EPG magnetisation pathways are characterised by their current phase state ($k$). This does not consider their full evolution history, and individual magnetisation pathways that ultimately end up in the same phase state may have experienced a different gradient waveform, $\overrightarrow{G}(t)$, over time. When considering time-*independent* diffusion systems, this leads to magnetisation pathways associated with different b-values in the same phase state ($k$) being combined (Fig. 3). For time-*dependent* diffusion systems, information about the specific gradient waveform, $\overrightarrow{G}(t)$, experienced by an individual magnetisation pathway is irretrievably lost (Fig. 3). Existing analytical representations of the DW-SSFP signal are similarly impacted.

When considering time-*independent* diffusion, it is possible to extend conventional EPG to investigate systems beyond free Gaussian diffusion. For example, the DW-SSFP signal under a diffusivity distribution $\rho \left(D\right)$ can be evaluated via numerical integration (${S}_{\text{DW}-\text{SSFP}}=\int \rho \left(D\right)\cdot EPG\left(\dots , D\right)dD$). To date, numerical EPG extensions to incorporate more sophisticated distributions (e.g. Watson^41^ and Bingham^42^ distributions) have not been explored. When considering time-*dependent* diffusion, it is not possible to use conventional EPG to characterise the measured signal, as the gradient waveform associated with each magnetisation pathways cannot be recovered. Existing analytical representations of the DW-SSFP signal are similarly impacted.

Below, I describe a framework that preserves information about the history of gradient waveforms experienced by magnetisation pathways to investigate time-*independent* and -*dependent* diffusion systems using DW-SSFP, with time-*dependence* characterised via the GPA^27,29,30^. Specifically, the framework can (i) estimate diffusion attenuation arising from time-*independent* and -*dependent* diffusion systems, (ii) incorporate existing biophysical models without the requirement of extensive derivations; (iii) translate defined DW-SSFP parameters into an interpretable measurement; and (iv) incorporate the impact of alternative gradient waveforms on the DW-SSFP signal. Analogous to conventional EPG providing a solution to the Bloch-Torrey equation, the proposed framework can be interpreted as a solution to the Bloch-Torrey equation averaged over sub-voxel microstructure arising from time-*independent* and -*dependent* diffusion systems.

### Overview

The proposed framework builds on EPG to distinguish pathways that have an identical phase ($k$) state, but different evolution histories. Importantly, whilst conventional EPG mitigates the challenge of dimensionality by combining magnetisation pathways with identical phase ($k$) states, here I mitigate the challenge of dimensionality by combining components of magnetisation pathways that evolve identically. This latter approach turns out to efficiently preserve information about the experienced gradient waveform of each magnetisation pathway, essential for incorporating time-*independent* and -*dependent* diffusion phenomena into DW-SSFP, with the current implementation suitable for investigating spoiled, periodic sequences. It can be alternatively interpreted as an extension to existing partition framework representations of DW-SSFP^11,25^.

The proposed approach requires us to first explicitly avoid the averaging of pathways with identical phase ($k$) states in EPG. Using the notation in Weigel^26^, I achieve this via:

1 $$\mathop {\underbrace {{S_{{\text{DW-SSFP}}} \left( {\tilde{F}_{0} } \right)}}_{(1)}}\limits_{{}} = \underbrace {{\frac{{1 - E_{1} }}{{1 - E_{1} \cdot \cos \alpha }}}}_{(2)} \cdot \underbrace {{\mathop \sum \limits_{{j = 0}}^{\infty } \left( {\underbrace {{{\varvec{A}}_{{{\varvec{ET}}}} { \circledast }{\varvec{A}}_{{{\varvec{ET}}}} { \circledast } \ldots {\varvec{A}}_{{{\varvec{ET}}}} { \circledast }}}_{{\text{j times}}}} \right)}}_{(3)}\underbrace {{\left[ {\begin{array}{*{20}c} { - i \cdot E_{2} \cdot e^{i\phi } \cdot \sin \alpha } \\ {i \cdot E_{2} \cdot e^{ - i\phi } \cdot \sin \alpha } \\ 0 \\ \end{array} } \right]}}_{(4)},$$

consisting of:

1. The DW-SSFP signal (sum of all ${\widetilde{F}}_{0}$ pathways)
2. Steady-state longitudinal magnetisation
3. Application of the RF-excitation (${\varvec{T}}$) & relaxation (${\varvec{E}}$) EPG operator^26^
4. Initialised magnetisation vector in the transverse plane [${\widetilde{F}}_{+1},{\widetilde{F}}_{-1},$ ${\widetilde{Z}}_{0}$]

where ${E}_{1/2}={e}^{-{T}_{1/2}/\text{TR}}$, $\circledast$ defines a pathway expansion operator (described below) and^26^:

2 $${{\varvec{A}}}_{{\varvec{E}}{\varvec{T}}}=\left[\begin{array}{ccc}{E}_{2}\cdot {\text{cos}}^{2}\frac{\alpha }{2}& {E}_{2}\cdot {e}^{2i\phi }\cdot {\text{sin}}^{2}\frac{\alpha }{2}& -{E}_{2}\cdot i\cdot {e}^{i\phi }\text{sin}\alpha \\ {E}_{2}\cdot {e}^{-2\mathit{i\phi }}\cdot {\text{sin}}^{2}\frac{\alpha }{2}& {E}_{2}\cdot {\text{cos}}^{2}\frac{\alpha }{2}& {E}_{2}\cdot i\cdot {e}^{-\mathit{i\phi }}\cdot \text{sin}\alpha \\ -{E}_{1}\cdot \frac{i}{2}\cdot {e}^{-i\phi }\cdot \text{sin}\alpha & {E}_{1}\cdot \frac{i}{2}\cdot {e}^{i\phi }\cdot \text{sin}\alpha & {E}_{1}\cdot \text{cos}\alpha \end{array}\right].$$

$\circledast$ in Eq. (1) can be interpreted as separating each magnetisation component mixed by the ${{\varvec{A}}}_{{\varvec{E}}{\varvec{T}}}$ operator, defining:

3 $${{\varvec{A}}}_{{\varvec{E}}{\varvec{T}}}\circledast {{\varvec{M}}}_{3\times m}=\left[\begin{array}{cccccccc}{{{\varvec{A}}}_{{\varvec{E}}{\varvec{T}}}}_{11}{M}_{11}& {{{\varvec{A}}}_{{\varvec{E}}{\varvec{T}}}}_{11}{M}_{12}& \dots & {{{\varvec{A}}}_{{\varvec{E}}{\varvec{T}}}}_{11}{M}_{1m}& {{{\varvec{A}}}_{{\varvec{E}}{\varvec{T}}}}_{12}{M}_{21}& {{{\varvec{A}}}_{{\varvec{E}}{\varvec{T}}}}_{12}{M}_{22}& \dots & {{{\varvec{A}}}_{{\varvec{E}}{\varvec{T}}}}_{13}{M}_{3m}\\ {{{\varvec{A}}}_{{\varvec{E}}{\varvec{T}}}}_{21}{M}_{11}& {{{\varvec{A}}}_{{\varvec{E}}{\varvec{T}}}}_{21}{M}_{12}& \dots & {{{\varvec{A}}}_{{\varvec{E}}{\varvec{T}}}}_{21}{M}_{1m}& {{{\varvec{A}}}_{{\varvec{E}}{\varvec{T}}}}_{22}{M}_{21}& {{{\varvec{A}}}_{{\varvec{E}}{\varvec{T}}}}_{22}{M}_{22}& \dots & {{{\varvec{A}}}_{{\varvec{E}}{\varvec{T}}}}_{23}{M}_{3m}\\ {{{\varvec{A}}}_{{\varvec{E}}{\varvec{T}}}}_{31}{M}_{11}& {{{\varvec{A}}}_{{\varvec{E}}{\varvec{T}}}}_{31}{M}_{12}& \dots & {{{\varvec{A}}}_{{\varvec{E}}{\varvec{T}}}}_{31}{M}_{1m}& {{{\varvec{A}}}_{{\varvec{E}}{\varvec{T}}}}_{32}{M}_{21}& {{{\varvec{A}}}_{{\varvec{E}}{\varvec{T}}}}_{32}{M}_{22}& \dots & {{{\varvec{A}}}_{{\varvec{E}}{\varvec{T}}}}_{33}{M}_{3m}\end{array}\right].$$

Specifically, whereas conventional EPG sums magnetisation components with identical $\tilde{F}_{k}$/$\tilde{Z}_{k}$ states (with each TR adding an additional set of states), the ${ \circledast }$ operator increases the number of states threefold per TR. This leads to a matrix of dimensions 3 × 3^m^, where the top/middle and bottom rows correspond to $\tilde{F}{ }$ and $\tilde{Z}$ states respectively.

Using Eqs. (1)–(3), I identify signal-contributing ($\tilde{F}_{0}$) magnetisation by separately tracking and storing the phase state evolution ($k\left( {{\text{TR}}_{1} } \right) \to k\left( {{\text{TR}}_{2} } \right) \to k\left( {{\text{TR}}_{3} } \right) \to \ldots$) of each magnetisation pathway via a shift operator ($\tilde{F}_{k} \to \tilde{F}_{k \pm 1} ;\tilde{Z}_{k} \to \tilde{Z}_{k}$), where $k$ can be positive or negative. The phase state evolution of each pathway that reaches an $\tilde{F}_{0}$ state is saved and used to synthetise its experienced gradient waveform, $\vec{G}\left( t \right)$(Fig. 3).

Equation (1) initialises all pathways in the transverse plane (4), with the contribution of steady-state longitudinal magnetisation modelled as a scalar constant (2). This initialisation builds on two principles:

1. Equilibrium longitudinal magnetisation ($\tilde{Z}_{0}$) experiences no gradient and is therefore insensitive to diffusion.
2. For periodic sequences, all $\tilde{Z}_{0}$ signal components (i.e. $\tilde{Z}_{0}$ components that arise from longitudinal recovery or magnetisation pathways that persist for several TRs in $\tilde{Z}_{0}$) will evolve in an identical manner once excited into the transverse plane, only distinguished by their signal amplitude prior to excitation. Their contribution can therefore be modelled as a scalar constant (equivalent to the sum of the signal amplitude of all magnetisation components in the $\tilde{Z}_{0}$ state), reducing the dimensionality of the problem.

Importantly, the above initialisation characterises any magnetisation component in an $\tilde{F}_{0}$ state as contributing to the measured signal independent of TR, unlike conventional EPG which characterises the measured signal as the amplitude of $\tilde{F}_{0}$ states at a specific TR. For example, after two TRs (one application of the ${\varvec{A}}_{{{\varvec{ET}}}}$ operator), a single $\tilde{F}_{0}$ state will arise (corresponding to a spin-echo pathway). After three TRs (two applications of the ${\varvec{A}}_{{{\varvec{ET}}}}$ operator), a second $\tilde{F}_{0}$ state will arise (corresponding to a stimulated-echo pathway). The sum of the signal amplitudes associated with $\tilde{F}_{0}$ states across all TRs (summation symbol in Eq. (1)) provides us with the total measured signal. To gain a further insight into the application of Eqs. (1)–(3) and these properties, I recommend the examples provided in the Supplementary Information.

Finally, note that Eq. (1) does not incorporate the effects of diffusion (i.e. there is no application of a diffusion operator, ${\varvec{D}}$). This enables us to integrate the effects of diffusion after identifying the amplitude of each signal-forming magnetisation pathway and their associated gradient waveforms, as described in the following sections.

### Pathway identification and storage

In practice, the number of pathways arising from Eq. (1) scales as 3^n^ (n = number of TRs). I previously motivated that for periodic sequences, $\tilde{Z}_{0}$ magnetisation components evolve in an identical manner after being excited into the transverse plane, enabling their individual contributions to be combined into a single scalar constant (Eqs. (1), (2)). Here, I build on this concept to further mitigate the challenge of dimensionality in Eq. (1) whilst preserving information about the experienced gradient waveform and signal amplitude of each magnetisation pathway by combining signal components with identical evolution histories.

To motivate this, Fig. 3 displays two magnetisation pathways (in blue and yellow) that have distinct diffusion properties and signal amplitudes, but identical phase states at the fourth TR. Importantly, following the fourth TR their magnetisation will evolve in an identical manner. Specifically, if we used Eq. (1) to model the evolution of the blue magnetisation pathway beyond the fourth TR, it would also characterise the evolution of the yellow magnetisation pathway. We can use this concept to create a memory-efficient approach to store the information required to synthesise any signal-forming magnetisation pathway.

Using this concept and the initialisation in (Eq. (1)), we can represent the evolution of all signal-forming pathways via:

4 $$\underbrace {{\tilde{F}_{ + 1/ - 1} \to \ldots \to }}_{{\{ {\text{i}}\} }}\quad \underbrace {{\tilde{F}_{0} \to \ldots \to }}_{{\{ {\text{ii}}\} }}\quad \underbrace {{\tilde{F}_{0} \to \ldots \to \tilde{F}_{0} }}_{{\{ {\text{ii}}\} }} \ldots$$

Equation (4) is built on four principles. Firstly, that only magnetisation in the $\tilde{F}_{0}$ state contributes to the measured DW-SSFP signal. Secondly, that a magnetisation pathway reaching an $\tilde{F}_{0}$ state will continue to evolve, with a subset of magnetisation components returning to the $\tilde{F}_{0}$ state at a future TR. Thirdly, that each instance of $\tilde{F}_{0}$ that arises during the evolution of Eq. (4) contributes to the measured DW-SSFP signal due to the initialisation in Eq. (1) (distinct from conventional EPG which characterises the measured signal as the amplitude of $\tilde{F}_{0}$ at a *specific* TR). Fourthly, that each $\tilde{F}_{0}$ instance has a distinct signal amplitude and experienced gradient waveform prior to reaching the $\tilde{F}_{0}$ state. This is an exact classification scheme, describing the evolution of all magnetisation pathways that reach an $\tilde{F}_{0}$ state.

Equation (4) describes magnetisation pathways as a function of two distinct components. The first component {i} describes the evolution of a pathway from an initial $\tilde{F}_{ + 1/ - 1}$ state to a signal-forming $\tilde{F}_{0}$ state. The second component {ii} describes the evolution of a pathway from the $\tilde{F}_{0}$ state to a further signal-forming $\tilde{F}_{0}$ state. Longer magnetisation pathways can be described by recursively appending components that are consistent with the definition of {ii}.

This description provides us with a memory-efficient approach to store the information required to synthesise any signal-forming magnetisation pathway. Specifically, all pathways that reach an $\tilde{F}_{0}$ state {i} will subsequently evolve in an identical manner, only distinguished by their signal amplitudes and experienced gradient waveforms prior to reaching the $\tilde{F}_{0}$ state (Fig. 3). If we therefore identify the signal amplitude and gradient waveform associated with all magnetisation pathways that evolve via $\tilde{F}_{ + 1/ - 1} \to \ldots \to \tilde{F}_{0}$ {i} and $\tilde{F}_{0} \to \ldots \to \tilde{F}_{0}$ {ii} once, we can use the construction in Eq. (4) to synthesise any signal-forming magnetisation pathway.

**Fig. 4:**
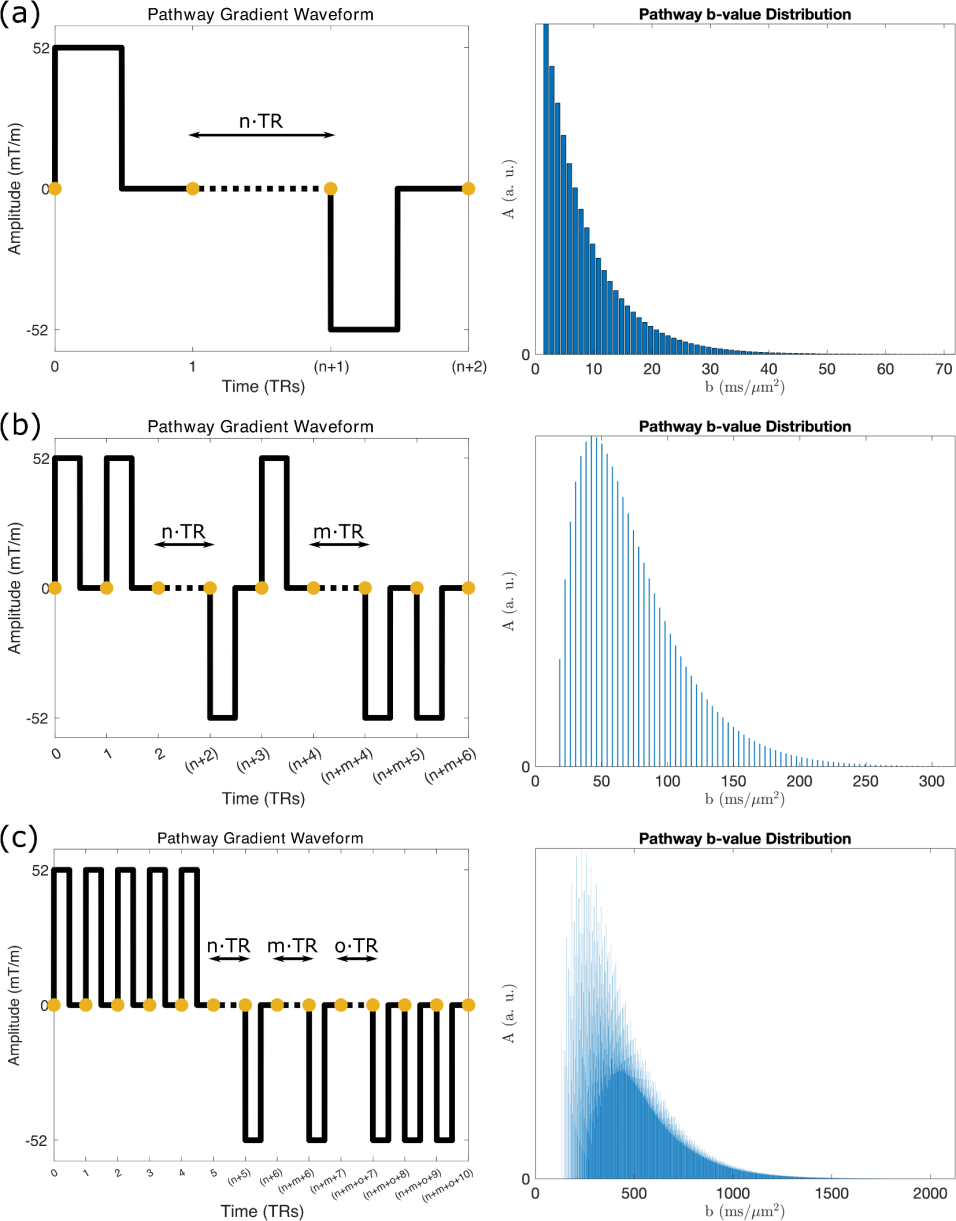
Example b-value distributions associated with dictionary components. Gradient waveforms experienced by three example dictionary entries (**a**–**c**, left) and their corresponding b-value distributions (**a**–**c**, right). (**a**) is the dictionary entry for all stimulated echoes. Here, the b-value distribution represents stimulated-echoes persisting for 1 to n TRs longitudinally. Dictionary entries with more than one longitudinal period (**b**,**c**) lead to more complicated distributions, arising in part due to different combinations of longitudinal period durations (i.e. different $n,\;m,\;o, \ldots$) achieving identical b-values, and distinct phase states associated with each longitudinal period. Properties of the three dictionary entries are provided in Supplementary Information Table S1. Yellow circles correspond to TR timings, with the spacing between yellow circles equivalent to a single DW-SSFP TR (spacing between a two RF pulses in Fig. 1c).

Using this approach I condense the information required to simulate all signal-forming pathway into two dictionaries. These dictionaries contain the signal amplitude and gradient waveform of all pathways evolving via either $\tilde{F}_{ + 1/ - 1} \to \ldots \to \tilde{F}_{0}$ {i} or $\tilde{F}_{0} \to \ldots \to \tilde{F}_{0}$ {ii}. I achieve this with Eq. (1), modelling all pathways that evolve from an initial $\tilde{F}_{ + 1/ - 1}$ state to an $\tilde{F}_{0}$ state once, and repeating the procedure from an initial $\tilde{F}_{0}$ state to create Dictionaries {i} and {ii}. We can use these dictionaries to estimate the properties of an arbitrary signal forming pathway by combining an entry in Dictionary {i} with one or more entries in Dictionary {ii}, multiplying signal amplitudes and appending gradient waveforms. I display the gradient waveforms associated with three example dictionary components in Fig. 4 (left column).

The dictionaries are further condensed by representing pathways that have identical gradient moments but experience a different number of longitudinal TRs ($\tilde{Z}_{k} \to \tilde{Z}_{k}$) as a single entry. For example, Fig. 4a (left) represents the gradient waveform of a single dictionary entry associated with all stimulated-echo pathways. This step is performed as we can exploit simple mathematical relationships between these pathways to rapidly estimate their properties, as described in the next section.

To prevent dictionaries from having an infinite number of entries, we can also define simple amplitude thresholds and limits on pathway durations. For the default parameters used in this manuscript (see “Methods”), dictionaries (i) and (ii) contained 23,474 and 46,948 entries respectively. They were generated in under 20 s on a personal laptop, requiring ~ 31 MB.

### Signal representation 1: time-independent diffusion

The first representation characterises the DW-SSFP signal in the time-*independent* regime by reparameterising the measured DW-SSFP signal as a b-value distribution. Specifically, whereas the diffusion attenuation arising from a DW-SE (or DW-STE) sequence can be accurately characterised with one b-value (equivalent to the b-value associated with a single SE or STE magnetisation pathway), the diffusion attenuation arising from a DW-SSFP sequence can be accurately described as a weighted sum of signal amplitudes associated with different b-values (arising from the signal amplitude and b-values associated with different signal-forming magnetisation pathways). A b-value distribution represents this reparameterization, displaying the relative amplitude of different signal components as a function of b-value.

To generate the b-value distribution associated with the measured DW-SSFP signal I first estimate the b-value distribution associated with each dictionary entry. For example, Fig. 4a (right) displays the b-value distribution associated with an entry in Dictionary {i} that represents stimulated-echo pathways.

Using the stimulated-echo entry as an example (Fig. 4a), I synthesise the b-value distribution by estimating the signal-amplitude and b-value associated with each instance of a different number of TRs between the two diffusion gradients via:

5 $$\begin{gathered} A\left( n \right) = A_{{{\text{init}}}} \cdot \left( {E_{1} \cdot \cos \;\alpha } \right)^{{\left( {n - 1} \right)}} \hfill \\ b\left( n \right) = \gamma^{2} G^{2} \delta^{2} \cdot k_{n}^{2} \cdot \left( {n - 1} \right) \cdot {\text{TR}} + b_{{{\text{init}}}} , \hfill \\ \end{gathered}$$

where $n$ defines an integer number of TRs between the two diffusion gradients (Fig. 4a), $k_{n}$ is the integer phase state associated with the longitudinal period of the stimulated-echo, $A_{{{\text{init}}}}$ is the signal amplitude the shortest ($n = 1$) stimulated-echo, and $b_{{{\text{init}}}}$ is the b-value associated with the shortest stimulated-echo (Supplementary Information Table S1). Each bar in Fig. 4a (right) corresponds to the signal amplitude and b-value associated with a different $n$.

We can generalise the above equation for arbitrary dictionary entries with several longitudinal periods (corresponding indices $n,\;m,\;o, \ldots$) by defining:

6 $$\begin{gathered} A\left( {n,\;m,\;o, \ldots } \right) = A_{{{\text{init}}}} \cdot \left( {E_{1} \cdot \cos \;\alpha } \right)^{{\left( {n - 1} \right) + \left( {m - 1} \right) + \left( {o - 1} \right) + \ldots }} \hfill \\ b\left( {n,\;m,\;o, \ldots } \right) = \gamma^{2} G^{2} \delta^{2} \cdot \left( {k_{n}^{2} \cdot \left( {n - 1} \right) + k_{m}^{2} \cdot \left( {m - 1} \right) + k_{o}^{2} \cdot \left( {o - 1} \right) + \ldots } \right) \cdot {\text{TR}} + b_{{{\text{init}}}} . \hfill \\ \end{gathered}$$

Modulation of the b-value by $k_{n,\;m,\;o, \ldots }^{2}$ reflects the increased q-value associated with longitudinal periods that have experienced repeat sensitisation to a diffusion gradient ($q = \gamma G\delta \cdot k_{n,m,o, \ldots } )$.

Figure 4 displays the b-value distribution of three example entries in Dictionary {i}, with their properties defined in Supplementary Information Table S1. As combinations of different longitudinal period durations (i.e. different $n,\;m,\;o, \ldots$) can share the same b-value, we can accurately represent the b-value distribution with reduced dimensionality.

Building on this approach, we can rapidly synthesise b-value distributions for different dictionary entries by exploiting simple relationships between them. For example, synthesising the b-value distribution of a dictionary entry with two longitudinal periods (example in Fig. 4b) requires estimation of the b-value and signal amplitude corresponding to every combination of longitudinal instances associated with different integer values of $m$ and $n$ (i.e. modelling all pathways associated with $n = 1$, $m = 1$ to $\infty$; $n = 2$, $m = 1$ to $\infty$; $n = 3$, $m = 1$ to $\infty$ etc.). This description is analogous to a convolution ($*$) of two dictionary entries with one longitudinal period (Supplementary Information Fig. S1), defining:

7 $$b_{{{\text{dist}}}} \left( {k_{n} ,\;k_{m} } \right) = b_{{{\text{dist}}}} \left( {k_{n} } \right)*b_{{{\text{dist}}}} \left( {k_{m} } \right) + b_{{{\text{init}}}} ,$$

where $b_{{{\text{dist}}}} \left( {k_{n} ,\;k_{m} } \right)$ is a b-value distribution corresponding to a dictionary component with two longitudinal periods (phase states $k_{n}$ and $k_{m}$), $b_{{{\text{dist}}}} \left( {k_{n} } \right)$ and $b_{{{\text{dist}}}} \left( {k_{m} } \right)$ are b-value distributions corresponding to a dictionary component with one longitudinal period (phase state $k_{n}$ or $k_{m}$), and $b_{{{\text{init}}}}$ is the b-value of the shortest gradient waveform associated with the dictionary entry with two longitudinal periods ($n = m = 1$). The above relationship can be generalised for dictionary entries with several longitudinal periods via:

8 $$b_{dist} \left( {k_{n} ,\;k_{m} , \ldots ,k_{y} ,\;k_{z} } \right) = b_{dist} \left( {k_{n} ,\;k_{m} , \ldots ,\;k_{y} } \right)*b_{dist} \left( {k_{z} } \right) + b_{{{\text{init}}}} .$$

The above relationships state that the shape of the b-value distribution associated with a dictionary entry is uniquely defined by (1) the number of distinct longitudinal periods ($n_{long}$) and (2) the phase state associated with each longitudinal period ($k_{n,m,o, \ldots }$). The only difference in the b-value distribution of dictionary entries with identical $n_{long}$ and $k_{n,m,o, \ldots }$ is associated with their b-value offset $b_{{{\text{init}}}}$ (Supplementary Information Fig. S2). The 23,474 and 46,948 entries in Dictionaries {i} and {ii} are associated with 680 unique b-value distribution shapes.

Once we have identified the b-value distribution associated with each entry in Dictionaries {i} and {ii} (Eq. (4) and surrounding text), we sum together the distributions per dictionary to create two b-value dictionary distributions, $b_{{{\text{dist}}_{{\left\{ {\text{i}} \right\}}} }}$ and $b_{{{\text{dist}}_{{\left\{ {{\text{ii}}} \right\}}} }}$. We can generate the final b-value distribution via a similar recursive convolution operation:

9 $$b_{{{\text{dist}}}} = b_{{{\text{dist}}_{{\left\{ {\text{i}} \right\}}} }} + b_{{{\text{dist}}_{{\left\{ {\text{i}} \right\}}} }} *b_{{{\text{dist}}_{{\left\{ {{\text{ii}}} \right\}}} }} + b_{{{\text{dist}}_{{\left\{ {\text{i}} \right\}}} }} *b_{{{\text{dist}}_{{\left\{ {{\text{ii}}} \right\}}} }} *b_{{{\text{dist}}_{{\left\{ {{\text{ii}}} \right\}}} }} + \ldots ,$$

where the different components in the above equation correspond to pathways that evolve via $\tilde{F}_{ + 1/ - 1} \to \ldots \to \tilde{F}_{ - 0}$; $\tilde{F}_{ + 1/ - 1} \to \ldots \to \tilde{F}_{ - 0} \to \ldots \to \tilde{F}_{ - 0}$; $\tilde{F}_{ + 1/ - 1} \to \ldots \to \tilde{F}_{ - 0} \to \ldots \to \tilde{F}_{ - 0} \to \ldots \to \tilde{F}_{ - 0}$ et﻿c.

**Fig. 5:**
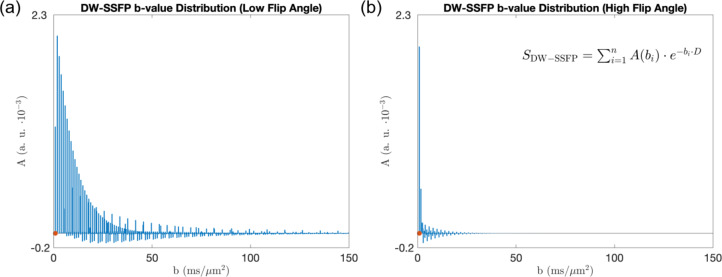
DW-SSFP b-value representation. By estimating the b-value and amplitude of each signal-forming pathway, the measured DW-SSFP signal can be represented as the weighted sum of signal fractions with different b-values ($A$ defined in Eq. (10)). Here I visualise this representation for DW-SSFP data acquired at a (**a**) low (20°) and (**b**) high (160°) flip angle, providing insights into how changing a DW-SSFP parameter translates into probing a given b-value regime. We can subsequently estimate the measured DW-SSFP signal by summing over the b-value range (x-axis), scaling the amplitudes with an appropriate biophysical model (Eq. (10)—inset equation corresponds to free Gaussian diffusion). The orange dots indicate the equivalent DW-SE b-value assuming identical gradient amplitudes and timings (setting ${\Delta } = {\text{TR}}$), equal to $0.836 \;{\text{ms}}/{\upmu \text{m}}^{2}$. Parameters based on post-mortem DW-SSFP investigations at 7T as described in Tendler et al.^32^ (defined in “Methods”). To explore how changing parameters influences the distributions, find the associated code here.

Figure 5 displays the final b-value distribution associated with the default investigation parameters in this manuscript (see “Methods”). It reparameterises the DW-SSFP signal as the weighted-sum of signal fractions with different b-values. It represents the information of over 10^18^ pathways, and was synthesised in under 15 s on a personal laptop. Note that pathways can have positive or negative signal amplitudes (Supplementary Information Fig. S3).

This representation provides insight into the b-values associated with DW-SSFP measurements. By changing the sequence parameters or sample properties, we observe a change to the relative-weighting of pathways with different b-values. Here, a reduced DW-SSFP flip angle corresponds to an increased signal fraction at higher b-values, lead to greater diffusion-weighting of the DW-SSFP signal (Fig. 2). It also demonstrates how DW-SSFP achieves strong diffusion-weighting, with the majority of signal arising from b-values that exceed those arising from a DW-SE with identical diffusion-encoding gradient waveforms and timings.

A powerful aspect of this representation is that it also enables us to directly translate existing time-*independent* models into DW-SSFP without deriving novel analytical forms. Specifically, we can use the b-value distribution to represent the measured DW-SSFP signal as:

10 $$S_{{\text{DW-SSFP}}} = \mathop \sum \limits_{i = 1}^{n} A\left( {b_{i} } \right) \cdot {\text{Model}}\left( {{\text{b}}_{i} } \right),$$

where $A\left( {b_{i} } \right)$ is the signal amplitude at each b-value (Fig. 5), $n$ is the number of b-values in the distribution and ${\text{Model}}\left( {b_{i} } \right)$ defines any time-*independent* model or representation. For example, for free Gaussian diffusion:

11 $${\text{Model}}\left( {{\text{b}}_{{\text{i}}} } \right) = e^{{ - b_{i} \cdot D}},$$

for a Tensor:

12 $${\text{Model}}\left( {{\text{b}}_{{\text{i}}} } \right) = e^{{ - b_{i} \hat{g}_{k} {\varvec{D}}\hat{g}_{k} }} ,$$

For the Standard Model of white matter^37^ (evaluated via e.g. NODDI^43^)

13 $${\text{Model}}\left( {{\text{b}}_{{\text{i}}} } \right) = {\text{NODDI}}\left( {b_{i} , \ldots } \right).$$

This is particularly helpful in DW-SSFP due to the complicated nature of its signal on both tissue and sequence parameters. When considering free Gaussian diffusion, Eq. (10) leads to the same result as conventional EPG or an analytical DW-SSFP model (Appendix 1).

Unlike previous partition framework implementations utilising the two-transverse^23,25^ or four-transverse^44^ period approximations of DW-SSFP, a key advantage of the proposed time-*independent* pathway framework is that it can incorporate higher-order signal components to accurately represent the DW-SSFP signal. This is particularly valuable when considering regimes where the T_2_ >> TR (Supplementary Information Fig. S4).

### Signal representation 2: time-dependent diffusion

In this work I investigate time-*dependent* diffusion phenomena via the Gaussian Phase Approximation^27,29,30^ (GPA). In contrast to a waveform’s b-value (which only considers the total gradient integral), the GPA explicitly incorporates information about the timings of the gradient waveform via the evolution of the q-vector, $q\left( t \right) = \gamma \smallint G\left( t \right)dt$. It reparameterises the estimated diffusion attenuation as a function of the encoding power applied at different temporal frequencies of the diffusion spectrum (Fourier transform of the velocity-autocorrelation function^27^) of the underlying microstructural system.

Using a similar approach to the previous section, I estimate the DW-SSFP signal via the GPA as:

14 $$S_{{\text{DW-SSFP}}} = \mathop \sum \limits_{i = 1}^{n} A_{i} \left( {T_{1} ,\;T_{2} ,\;\alpha ,\;{\text{TR}}} \right) \cdot e^{{\mathop \int \limits_{ - \infty }^{\infty } \widetilde{{q_{i} }}\left( \omega \right)\tilde{D}\left( \omega \right)\widetilde{{q_{i} }}\left( { - \omega } \right)d\omega }} ,$$

where $A_{i} \left( {T_{1} ,\;T_{2} ,\;\alpha ,\;{\text{TR}}} \right)$ is the amplitude of a signal forming pathway, $\widetilde{{q_{i} }}\left( \omega \right)$ is the Fourier transform of the pathway’s q-vector, $n$ is the number of pathways, and $\tilde{D}\left( \omega \right)$ is the diffusion spectrum of the microstructural system.

**Fig. 6:**
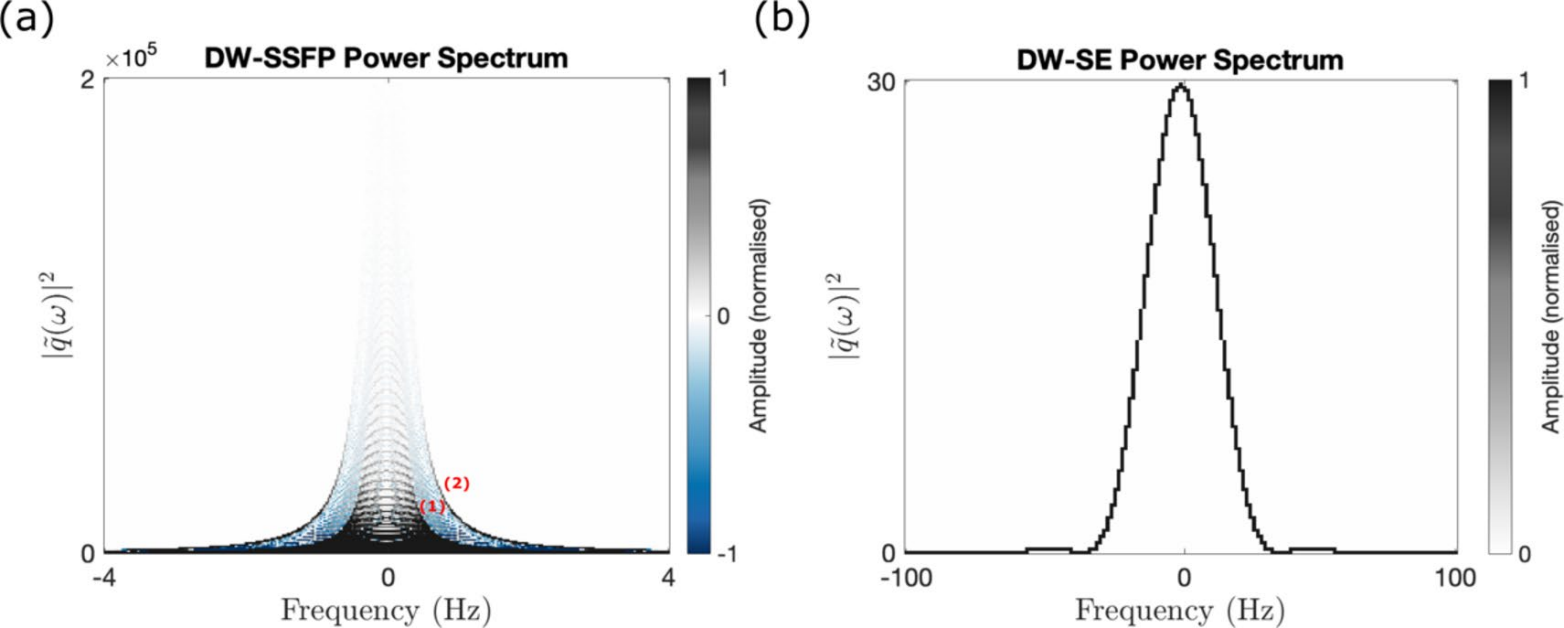
DW-SSFP power spectrum representation. (**a**) By estimating $|\widetilde{{q_{i} }}\left( \omega \right)\left.\right|^{2}$ and the amplitude of each signal-forming pathway, the measured DW-SSFP signal is represented as a power spectrum density plot. Here, image colour represents the signal amplitude contributed to a given regime, with DW-SSFP pathways associated with both positive and negative amplitudes (Supplementary Information Fig. S3). (**b**) displays the equivalent power spectrum for a DW-SE sequence using identical gradient timings (setting ${\Delta } = {\text{TR}}$). For DW-SSFP, $|\widetilde{{q_{i} }}\left( \omega \right)\left. \right|^{2}$ spans a narrower frequency range with substantially higher amplitudes, where both x and y axes in (**a**) and (**b**) span different extents. ‘Contours’ in (**a**) (labelled 1 and 2) correspond to pathways experiencing two and four periods in the transverse plane. For equivalent spectrums at different flip angles, see Supplementary Information Fig. S5. (**a**) displays $|\widetilde{{q_{i} }}\left( \omega \right)\left. \right|^{2}$ for pathways that experience up to four periods in the transverse plane. Parameters based on post-mortem DW-SSFP investigations at 7T as described in Tendler et al.^32^ (defined in “Methods”). To explore how changing parameters influences the power spectrum, find the associated code here.

Figure 6 reparameterises the DW-SSFP signal as a as density plot of gradient power-spectra. Like the b-value representation (Fig. 5), by changing DW-SFP sequence parameters or sample properties we observe a change the relative amplitude and width of $|\widetilde{{q_{i} }}\left( \omega \right)\left. \right|^{2}$ (Supplementary Information Fig. S5). This representation demonstrates that the DW-SSFP signal is typically strongly concentrated in a narrow range of the frequency spectrum when compared to a conventional DW-SE.

Unlike the highly compressible signal representation for time-*independent* diffusion, there is little degeneracy of $\tilde{q}\left( \omega \right)$ for pathways that contribute to the measured DW-SSFP signal. For the time-*dependent* diffusion representation I use the dictionaries (“Theory”: “Pathway identification and storage”) to explicitly generate the full gradient waveform and signal amplitude of *each* individual signal-forming pathway. I achieve this by recursively appending the gradient waveforms from the Dictionary {i} ($\tilde{F}_{ \pm 1} \to \ldots \to \tilde{F}_{0}$) and {ii} ($\tilde{F}_{0} \to \ldots \to \tilde{F}_{0}$) and multiplying their signal amplitudes. I identify the location of each longitudinal period (Fig. 4—dashed lines) and extend each gradient waveform for different periods in the longitudinal plane, scaling the pathway signal amplitudes by $(E_{1} \cdot \cos \alpha )^{{\left( {n - 1} \right) + \left( {m - 1} \right) + \left( {o - 1} \right) + \ldots }}$, where $n,\;m,\;o \ldots$ correspond to the number of TRs associated with each longitudinal instance (Eq. (6)).

A key challenge of the time-*dependent* pathway framework is the requirement to explicitly incorporate the full gradient waveform and signal amplitude of each pathway separately, leading to long computational times. To address this, in this work I limited investigations to pathways that persist for up to four TRs in the transverse plane (four transverse-period approximation). This approximation corresponds to ~ 99% of the measured signal for the investigated parameter regime (Supplementary Information Fig. S6), corresponding to ~ 275,000 pathways. In the results section I present findings utilising the time-*dependent* pathway framework to investigate a restricted cylinder system with varying radius. Specifically, the system consists of a single restricted cylinder where the longitudinal axis is oriented perpendicular to the applied diffusion gradient. For simulations, only the signal from the ‘intra-cylinder’ compartment (i.e. within the cylinder) were considered. Note that this system is distinct from the neurite compartment in the Standard Model of white matter, which assumes measurements are insensitive to radius size by modelling cylinders as ‘sticks’ with no transverse diffusion coefficient (i.e. $D_{{ \bot {\text{Cyl}}}} = 0$).

### Alternative gradient waveforms

We can use the above frameworks to investigate the impact of alternative gradient waveforms on the DW-SSFP signal by explicitly integrating the waveform shape into the investigation. This can be achieved without additional coding or analytical derivations.

**Fig. 7:**
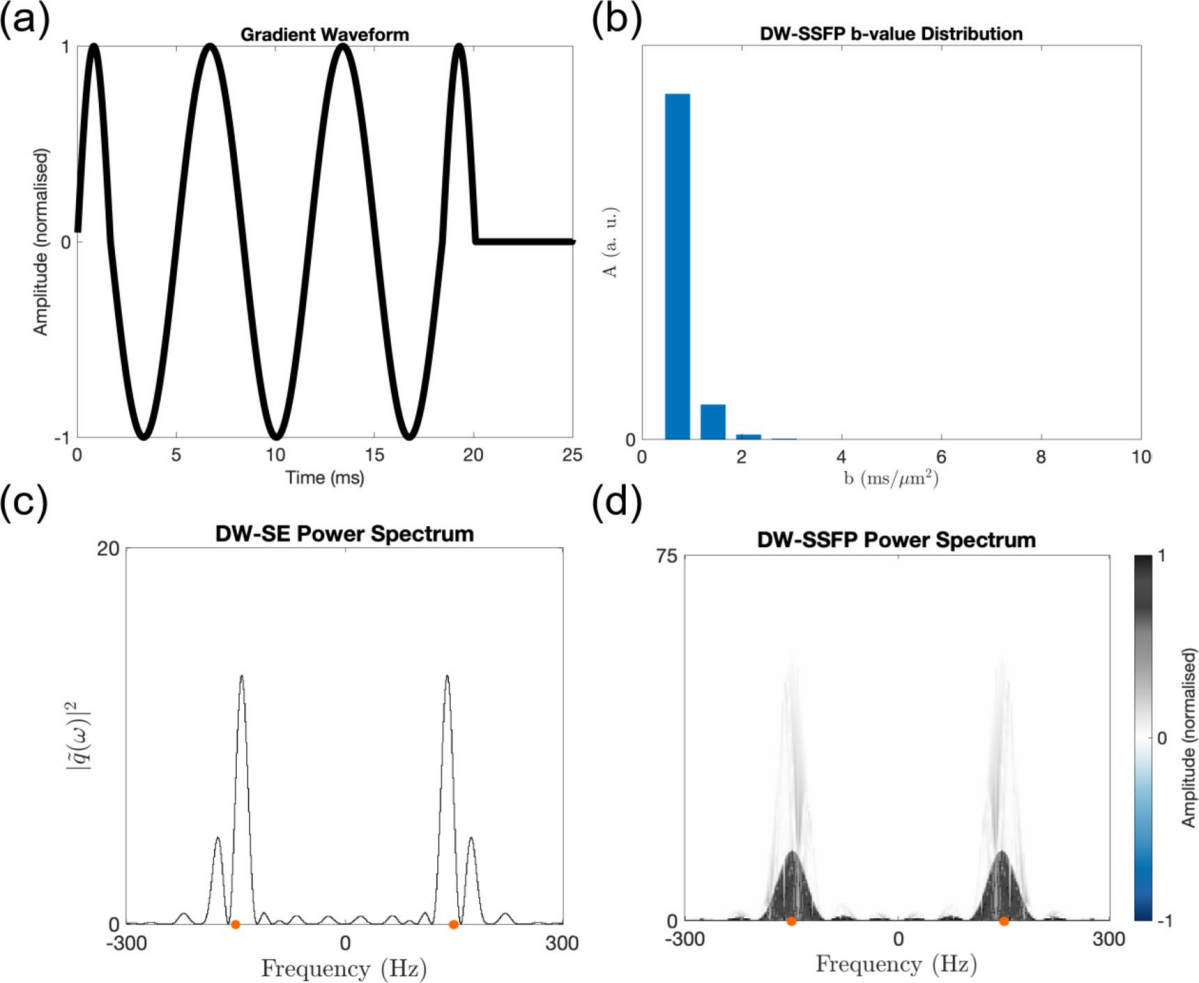
DW-SSFP with oscillating gradients. Using the balanced sinusoidal gradient waveform in (**a**) (equivalent to Fig. 1d in Aggarwal et al.^45^—oscillation frequency = 150 Hz), we can characterise the DW-SSFP signal with oscillating gradients as a b-value distribution (**b**). Here, each peak corresponds a $2 \cdot n$ multiple of the waveforms b-value ($b = 0.35 \;{\text{ms}}/{\upmu \text{m}}^{2}$). (**c**) represents the power-spectrum for a DW-SE using the gradient waveform in (**a**) (analogous to Figure 1e in Aggarwal et al.^45^), with peaks centred around ± 150 Hz (orange dots). By estimating $|\widetilde{{q_{i} }}\left( \omega \right)\left. \right|^{2}$ and the amplitude of each signal-forming pathway, the DW-SSFP power spectrum density plot (d) displays similar characteristics, with peak amplitudes centred around ± 150 Hz (orange dots). This shows that DW-SSFP is capable of probing different frequency components of the diffusion spectrum, as reflected by deviation of central peak of the power spectrum plot away from 0 Hz. Parameters based on Aggarwal et al.^45^ (see “Methods”). (**a**) represents the experienced gradient waveform in a single TR of the DW-SSFP sequence (equivalent to the timing between two RF pulses in Fig. 1c). (**d**) displays $|\widetilde{{q_{i} }}\left( \omega \right)\left. \right|^{2}$ for pathways that experience up to four periods in the transverse plane. To explore how changing parameters influences the distributions with oscillating gradients, find the associated code here.

Figure 7 displays the predicted DW-SSFP b-value distribution and power-spectrum with oscillating gradients (sinusoidal waveform and parameters based on Aggarwal et al.^45^). Here, each TR of the DW-SSFP sequence contains a single oscillating gradient (Fig. 7a), in contrast to a conventional DW-SE sequence which contains a pair of oscillating gradients per TR. This is consistent with the definition of DW-SSFP and DW-SE for unbalanced gradients, as motivated in the sequence diagrams in Fig. 1.

The b-value distribution (Fig. 7b) arising from the time-in*dependent* framework provides an efficient description of the DW-SSFP signal, condensing the entire measurement into a few discrete signal components with different b-values (scalar multiples of the b-value per TR). The distribution corresponds to the fractions of signal that experience $2 \cdot n$ TRs in the transverse plane (integer $n$).

The time-*dependent* pathway framework demonstrates that DW-SSFP with integrated oscillating gradients can probe different frequency components of the diffusion spectrum (Fig. 7c,d), here centred around the oscillation frequency of the sinusoidal gradient waveform ($f = 150\; {\text{Hz}}$). The addition of oscillating gradients into the DW-SSFP sequence is therefore analogous to the implementation of oscillating gradients for conventional diffusion MRI sequences.

Note that the DW-SSFP sequence incorporates an unbalanced gradient per TR to prevent banding artefacts arising from off-resonance effects. To address this for the balanced oscillating gradients waveform presented here, we can introduce an independent, small unbalanced gradient; slightly unbalance the oscillating waveform^46^; or add a small vertical offset between positive and negative gradient lobes. A sufficiently small, unbalanced gradient typically leads to negligible diffusion-weighting and can be integrated into the proposed framework.

### Accuracy of DW-SSFP approximations

The b-value distributions arising from the time-in*dependent* framework with oscillating gradients (Fig. 7b) can alternatively be used to assess the accuracy of DW-SSFP approximations. Specifically, a commonly used alternative model of the DW-SSFP signal is the ‘two-transverse’ period approximation^23,25^, only considering pathways that persist for up to two TRs in the transverse plane. The accuracy of this approximation is reflected in the difference between the left-most component of the b-value distribution with oscillating gradients (Fig. 7b, reflecting the total signal from all pathways experiencing two transverse TRs), and the remaining signal from all other components. This approach can be extended for alternative simplified signal models (e.g. four-transverse period approximation^44^).

The b-value distributions can also be used to provide insights into which parameter regimes approximations are most precise. For example, the b-value distributions demonstrate that the two-transverse period approximation is *least* accurate at very low (< 5°) and very high flip (> 160°) angles (Supplementary Information Fig. S7). This reflects the contribution of stimulated-echo pathways that persist for several periods in the transverse plane (very low flip angles) and spin-echo pathways that experience repeated pairs of dephasing and rephasing (very high flip angles).

## Results

**Fig. 8:**
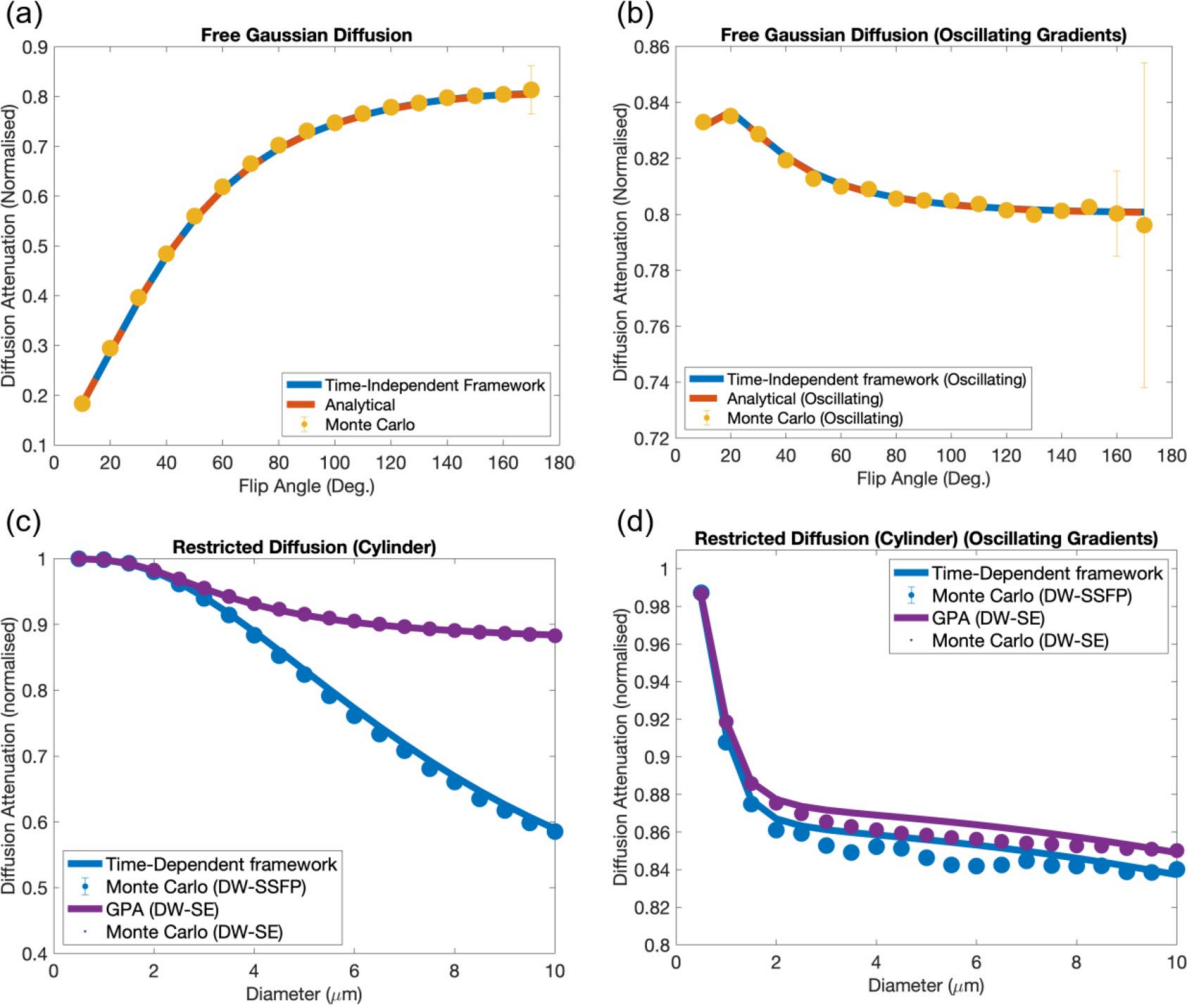
DW-SSFP signal estimation. The proposed time-*independent* framework demonstrates excellent agreement with analytical DW-SSFP signal models and complementary Monte Carlo simulations across a range of flip angles for (**a**) conventional (unbalanced diffusion gradient) and (**b**) oscillating DW-SSFP sequences. The time-*dependent* framework characterising diffusion in a single cylinder of varying radius gives good agreement to Monte Carlo simulations for conventional DW-SSFP (**c**), conceptually achieving higher diffusion attenuation compared to a DW-SE with matched diffusion encoding waveforms and timings. When considering oscillating gradients (**d**), the time-*dependent* framework gives similar accuracy to Monte Carlo simulations when compared to the DW-SE, with DW-SSFP predicting a small increase in signal attenuation. Parameters based on Tendler et al.^32^ for conventional gradients (see “Methods”) and Aggarwal et al.^45^ for oscillating gradients (see “Methods”). Equivalent representation of restricted diffusion for the DW-SE sequence implemented based on the Gaussian Phase Approximation^27,29,30^ using the proposed time-*dependent* framework. Monte Carlo simulation error bars (defined as the signal standard deviation from the final 10 TRs) are too small to be visualised for most datapoints. Note that the y axes span different extents in each plot. To explore how changing parameters influences these relationships, find the associated code here.

Figure 8a estimates the DW-SSFP signal for free Gaussian diffusion as a function of flip angle using (1) the proposed time-*independent* pathway framework, (2) an analytical DW-SSFP signal model^33^ (Appendix 1) and (3) Monte Carlo simulations. Results demonstrate excellent agreement between the three approaches, with conventional DW-SSFP demonstrating an expected increase in diffusion attenuation with reduced flip angle^24^ (Fig. 5).

Figure 8b repeats the investigation with oscillating gradients, finding that the estimated diffusion attenuation is relatively insensitive to changes in flip angle. Here, increasing the flip angle initially leads to a reduction in diffusion attenuation, with a subsequent rise in attenuation at higher flip angles. This can be interpreted by looking at the evolution of the b-value distributions (Supplementary Information Fig. S7). Specifically, the relative fraction of signal that experiences $2 \cdot {\text{n}} \cdot {\text{TRs}}$ in the transverse plane is almost identical at very low (1°) and very high flip (179°) angles, despite differences in the pathways that predominantly contribute to the measured signal (see “Theory”: “Accuracy of DW-SSFP approximations”). Excellent agreement is again found between the three investigative approaches.

Figure 8c investigates a restricted cylinder system with varying radius, comparing the proposed time-*dependent* pathway framework to Monte Carlo simulations, with good agreement between the two approaches. Here, the average difference in attenuation between the proposed time-*dependent* framework and Monte Carlo simulations is 0.9% for the investigated parameters with DW-SSFP (0.09% for the DW-SE comparison). Comparison to a DW-SE sequence with matched gradient waveforms and timings demonstrates that DW-SSFP conceptually achieves higher levels of diffusion attenuation for the same restriction system.

Figure 8d displays a similar investigation with oscillating gradients. Here, DW-SSFP achieves a small conceptual increase in diffusion attenuation compared to a DW-SE sequence. Here, the average difference in attenuation between the proposed time-*dependent* framework and Monte Carlo simulations is 0.7% for the investigated parameters (0.5% for the DW-SE comparison).

**Fig. 9:**
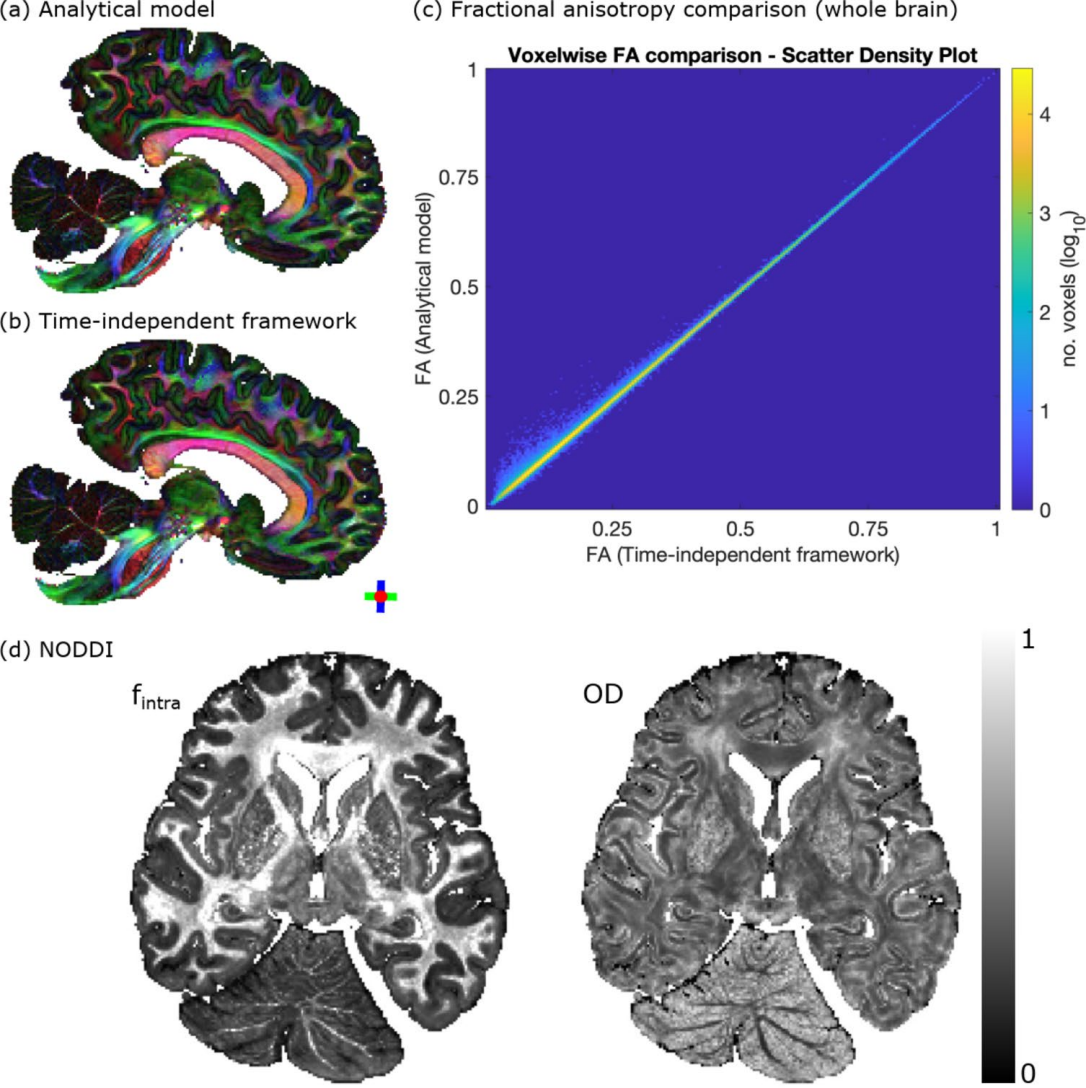
Experimental analysis. Comparison of tensor estimates derived from experimental post-mortem DW-SSFP data (whole human brain) based on the analytical DW-SSFP model^33^ (Appendix 3) and proposed time-*independent* pathway framework. (**a**,**b**) Fractional anisotropy (FA) modulated principal diffusion direction maps give excellent spatial agreement. (**c**) Scatter density plot of voxelwise FA estimates (whole brain) demonstrates the accuracy of the proposed time-*independent* pathway framework (Pearson correlation coefficient R = 0.9992). (**d**) Post-mortem NODDI estimates of intracellular volume fraction ($f_{intra}$) and orientation dispersion ($OD$) derived using the time-*independent* pathway framework. Details of experimental acquisition and processing provided in the “Methods”.

Figures 9a,b compare a fitted diffusion tensor derived from experimental DW-SSFP MRI data acquired in a post-mortem human brain (time-*independent* pathway framework) and an analytical DW-SSFP model^33^ with integrated tensor (Appendix 3). Voxelwise analysis (Fig. 9c) demonstrates excellent agreement between the two approaches (Pearson correlation coefficient R = 0.9992). Figure 9d displays proof of principle estimates of the intracellular volume fraction ($f_{intra}$) and orientation dispersion ($OD$) by incorporating NODDI into the time-*independent* pathway framework. The resulting $f_{intra}$ estimates demonstrate a consistently higher intracellular volume fraction within white matter. The $OD$ estimates display limited contrast between white matter and grey matter in the cerebrum, with the greatest distinction within the cerebellum.

**Fig. 10:**
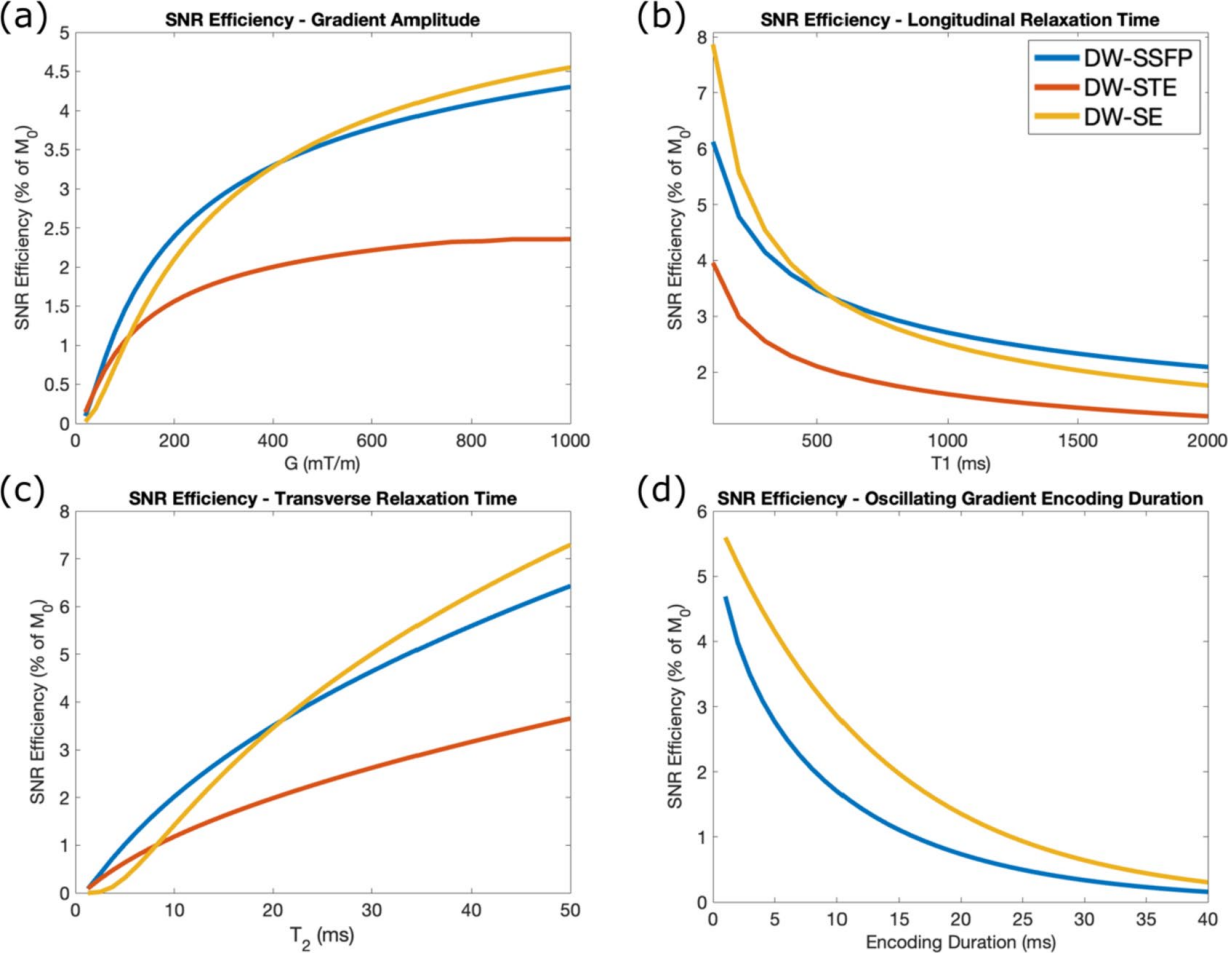
SNR-efficiency. SNR-efficiency estimation for DW-SSFP, DW-SE and DW-STE at a target b-value ($b = 10 {\text{ms}}/{\upmu \text{m}}^{2}$) as a function of gradient strength (**a**), T_1_ (**b**) and T_2_ (**c**). DW-SSFP predicts higher SNR-efficiency when compared to the DW-STE sequence across all investigated regimes, with the exception of very low gradient strengths. (**a**) The SNR-efficiency performance of DW-SSFP is reduced when compared to the DW-SE with increased gradient strength. (**b**) and (**c**) investigate the relationship between SNR-efficiency and relaxation regime at G = 1000 mT/m (rightmost point in (**a**)). DW-SSFP predicts higher SNR-efficiency with increased T_1_ and reduced T_2_ when compared to the DW-SE, motivating its use at ultra-high field. When considering oscillating gradients (**d**), DW-SSFP achieves reduced SNR-efficiency when compared to the DW-SE for matched encoding duration (per gradient). Simulations based on mean estimates of T_1_, T_2_ and D from a cohort of post-mortem brains assuming free diffusion. Effective b-value for DW-SSFP based on matched levels of diffusion attenuation (corresponding to $b = 10 \;{\text{ms}}/{\upmu \text{m}}^{2}$ for DW-SE). For full information of implementation, see “Methods”. To explore how changing parameters influences these relationships, find the associated code here.

Finally, Fig. 10a–c compares the theoretical SNR-efficiency properties of DW-SSFP, DW-SE and DW-STE as a function of gradient strength (Fig. 10a), T_1_ (Fig. 10b) and T_2_ (Fig. 10c) for matched attenuation levels (corresponding to $b = 10 {\text{ms}}/{\upmu \text{m}}^{2}$ for DW-SE). The relative SNR benefits of DW-SSFP reduce as a function of gradient strength when compared to the DW-SE (Fig. 10a), with DW-SE estimating improved SNR-efficiency at G > 420 mT/m in the investigated parameter regime. When considering the maximum investigated gradient strength (G = 1000 mT/m), DW-SSFP predicts improved SNR-efficiency in tissues with long T_1_ and short T_2_ (Fig. 10b,c), the relaxation conditions associated with ultra-high field. Combined with distortion-free outputs (DW-SSFP does not require an EPI readout), DW-SSFP may offer considerable benefits for ultra-high field microstructural investigations.

For oscillating gradients, DW-SSFP predicts reduced SNR-efficiency as a function of encoding duration when compared to the DW-SE in the investigated regime (Fig. 10d). Whilst DW-SSFP can theoretically achieve higher diffusion-weighting with matched gradient waveforms (Fig. 8b,d), these benefits could be mitigated by increasing the encoding period of the DW-SE sequence (e.g. having multiple repeats of a gradient waveform at a target frequency). These findings are broadly preserved when considering relaxation regimes found in post-mortem and in vivo tissue (Supplementary Information Fig. S8). The potential uses of DW-SSFP with oscillating gradients may be limited to investigational regimes where image distortions are particularly prominent.

## Discussion

### Interpreting DW-SSFP measurements

My framework shows that individual DW-SSFP measurements are equivalent to a distribution of signals summed across different b-values or $\tilde{q}\left( \omega \right)$ profiles (Figs. 5 and 6). This is in direct contrast to conventional diffusion MRI sequences that perform measurements at a single b-value or a single $\tilde{q}\left( \omega \right)$ profile. Changes in DW-SSFP parameters modify the relative weighting of b-values/$\tilde{q}\left( \omega \right)$ profiles that contribute to the measured signal (Fig. 5 and Supplementary Information Fig. S5). This can be considered the DW-SSFP equivalent of performing measurements at different b-value ‘shells’ or $\tilde{q}\left( \omega \right)$ profiles, providing an experimental basis to characterise tissue microstructure.

Figures 5 and 6 additionally demonstrate that conventional DW-SSFP samples high b-value regimes and a narrow band of the diffusion spectrum compared to conventional diffusion imaging sequences. The time-*dependent* pathway framework demonstrates that DW-SSFP with oscillating diffusion gradients can probe different frequency components of the diffusion spectrum (Fig. 7d), demonstrating a symmetric averaging of the power spectrum profile around the target frequency. Here, individual $\tilde{q}\left( \omega \right)$ profiles are centred around the target frequency with small changes in their distribution shape, leading to a broad, Gaussian-like function.

The sequence parameters of DW-SSFP provide additional dimensions to probe tissue microstructure compared to conventional sequences that alter the diffusion weighting through explicit changes to the diffusion-encoding module (i.e., q-value or diffusion time, Δ). For example, Fig. 8a,b demonstrate that we can achieve different levels of diffusion weighting by solely changing the acquisition flip angle, with no further changes to sequence timings. This effect can now be directly linked to modifications in the b-value distribution or power spectrum (Figs. 5, 6, 7). The observation of flip-angle dependent diffusion weighting agrees with previous experimental work with DW-SSFP data acquired at multiple flip angles^24,32^ and EPG theory^36^.

Several attempts have been previously made to define a single effective b-value in steady-state diffusion imaging to facilitate comparisons with conventional diffusion sequences^12,24,46^. However, these b-value estimates have either (1) been derived based on estimated diffusion properties of the investigated system^24^ and/or (2) approximated the measured DW-SSFP signal^12,14^. The time-*independent* pathway framework shows that you cannot characterise the DW-SSFP signal using a single effective b-value (${\text{b}}_{{{\text{eff}}}}$), as there is no general solution for Eq. (10) where $\mathop \sum \nolimits_{i = 1}^{n} A\left( {b_{i} } \right) \cdot {\text{Model}}\left( {{\text{b}}_{i} } \right) = A_{0} \cdot {\text{Model}}\left( {{\text{b}}_{{{\text{eff}}}} } \right)$. However, by re-formulating in terms of a b-value distribution, we can characterise and interpret the measured signal with extremely high precision.

### Characterising tissue microstructure with DW-SSFP

My investigation finds that the signal-forming mechanisms of DW-SSFP predict conceptually higher levels of diffusion attenuation in small radii cylinders compared to a DW-SE sequence with identical gradient waveforms and timings (Fig. 8c). The SNR-efficiency properties of DW-SSFP (Fig. 10a–c) reflects its potential for microstructural imaging when considering ultra-high field systems. Specifically, the theoretical SNR properties of DW-SSFP predict higher SNR-efficiency under matched diffusion attenuation when considering tissues with long T_1_ and short T_2_. These properties are reflected in the experimental dataset (Fig. 9), where DW-SSFP achieves high SNR diffusivity estimates in the challenging imaging environment of fixed post-mortem tissue at 7T. Here the sequences high SNR-efficiency and strong diffusion-weighting address the low diffusivity^47^ and short-T_2_^48^ of fixed samples.

A key advantage DW-SSFP is that it acquires diffusion images with low or negligible distortions. Specifically, the short TR of DW-SSFP is compatible with a single-line readout, with no distortion correction applied to the experimental data in Fig. 9 (three-line readout). At ultra-high field, increased distortions and short T_2_^*^ are particularly challenging when considering the EPI readouts associated with DW-SE. This suggests that DW-SSFP may still offer benefits for microstructural imaging in regimes where it achieves moderate reductions in SNR-efficiency when compared to the DW-SE.

In addition, whilst the signal-forming mechanisms of DW-SSFP are distinct, acquired datasets are compatible with several conventional preprocessing approaches. For example, noise floor biases arising from coil-combined data with low-SNR have been previously observed in DW-SSFP data^32^, and corrected using conventional noise-floor correction techniques^49^. DW-SSFP datasets are also compatible with modern complex-domain denoising methods (e.g. NORDIC^50^), and have been recently implemented to improve the SNR of DW-SSFP data acquired in post-mortem tissue^51,52^. A notable exception for DW-SSFP is preprocessing arising from subject motion, which leads to a distinct form of signal corruption^13^, requiring specialised methods to address. Broadly, this limits current DW-SSFP investigations to post-mortem or low-motion regimes.

When considering oscillating gradients, DW-SSFP is predicted to achieve a theoretical increase in diffusion attenuation (Fig. 8b,d). This increase in diffusion attenuation is strongly dependent on the T_2_ and TR of the sample, where an increased T_2_ relative to the TR will lead to more high-signal pathways with that experience repeat sensitisation to gradient pairs. However, the relative SNR-efficiency of DW-SSFP is reduced when compared to the DW-SE (Fig. 10d and Supplementary Information Fig. 8). Note that these observations do not encompass balanced steady-state sequences (e.g. TRUFI) with integrated oscillating gradients, which have not been investigated here.

The SNR-efficiency calculations presented here represent conceptual estimations of SNR-efficiency. They are based on simulations incorporating free Gaussian diffusion, and have not incorporated the impact of sequence dead time, readout duration limits, gradient duty cycle or T_2_^’^ decay. These properties are expected to vary depending on the precise implementation of the imaging sequence and properties of the MR system. For example, the short TR of the DW-SSFP sequence can lead to a high duty cycle arising from repeated application of the diffusion gradients in a short time interval, mitigated by a single-line readout which reduces gradient load across a TR. This contrasts with the DW-SE, with a reduced relative duty cycle contribution from diffusion gradients spaced over a longer TR, but additional contributions from a gradient-intensive EPI readout. From the context of post-mortem imaging, this can limit the maximum achievable gradient strength applicable, in addition to sample heating dependent on the specific sequence and implemented parameters.

### Time-independent pathway framework

The time-*independent* framework demonstrates excellent agreement to analytical solutions and Monte Carlo simulations (Fig. 8a,b). It provides high accuracy in comparison to existing approximation models of the DW-SSFP signal when T_2_ >> TR (Supplementary Information Fig. S4).

The time-*independent* framework exploits degeneracies in b-values to rapidly characterise the DW-SSFP signal. As the b-value distributions have relatively low dimensionality, we can pre-compute and store distributions to facilitate fitting of time-*independent* models or representations to data. This approach was utilised for the tensor and NODDI^43^ estimation in Fig. 9 (see “Methods”), demonstrating the utility of the framework as both a visualisation and parameter estimation tool. The experimental post-mortem comparison with diffusion tensor estimated parameters with excellent agreement to an analytical form (Fig. 9a–c), demonstrating the accuracy of the proposed method.

A key feature of the time-*independent* pathway framework is that it can be extended to incorporate more sophisticated models or representations based around compartments characterised by Gaussian diffusion without the requirement of extensive analytical derivations (Eqs. (10)–(13)). This is demonstrated via the integration of NODDI^43^ with experimental DW-SSFP data (Fig. 9d), where no analytical form currently exists. However, the accuracy of the derived NODDI parameters is currently unclear. This challenge arises due to (1) the additional complexity of performing NODDI in fixed post-mortem tissue^53^, and (2) whether DW-SSFP measurements can be approximated as time-*independent* in the investigated experimental regimes. Specifically, as DW-SSFP sensitises the signal to a wide range of b-values simultaneously in a single measurement, whether the assumption of time-independence holds across different parameter spaces. This was not considered when acquiring the experimental DW-SSFP data used in this manuscript, which formed part of a larger, separate project^31^, and remain the subject of future investigation.

The time-*independent* framework also provides context to identify biophysical models or representations that are suitable for DW-SSFP investigations. For example, a diffusion kurtosis signal representation^54^ breaks down in the high b-value range, which may correspond to a considerable fraction of the measured signal in a DW-SSFP measurement (Fig. 5a). The b-value distribution representation can therefore facilitate the identification of parameter regimes where a specific model or representation is valid, or alternatively inform model selection for microstructural investigations.

Note that a single component of the DW-SSFP b-value distribution does not correspond to signal associated with the same diffusion time. Here, changes along the x-axis may correspond to changes in the number of gradient pairs experienced by a magnetisation pathway, a change in the maximum amplitude of the diffusion gradient experienced over several TRs, or a longer effective diffusion time. If specific information about the gradient amplitude and timings are required for a given biophysical model, the time-*dependent* pathway framework, which preserves this information, is recommended.

### Time-*dependent* pathway framework

The proposed time-*dependent* framework provides a very general way to integrate existing models or representations incorporating time-*dependent* effects that (1) does not require analytical solutions and (2) provides visualisation of the diffusion spectrum regimes being investigated. These visualisations may support the identification of parameter spaces and novel gradient waveforms to probe distinct components of the diffusion spectrum (Figs. 6, 7).

The proposed time-*dependent* framework is based on the GPA^29^, which is typically considered valid in short and long diffusion time regimes. As a single DW-SSFP measurement combines multiple $\tilde{q}\left( \omega \right)$ profiles simultaneously (Figs. 6, 7), a subset of pathways that contribute to the measured DW-SSFP signal may not be well approximated by the GPA. Despite this, results indicate that the time-*dependent* pathway framework gives good agreement to Monte Carlo simulations when investigating the parameter regimes and sample properties utilised in previous experimental DW-SSFP work^32^ (Fig. 8c,d), with average errors of below 1%. Whilst I have not performed extensive investigations of the time-*dependent* pathway framework beyond the parameter regime explored in this article, the time-*dependent* pathway framework is not limited to the GPA, and could be readily extended to more advanced representations of the diffusion MRI signal^55^.

A key limitation of the time-*dependent* pathway framework is that each signal-forming pathway needs to be characterised separately to estimate diffusion attenuation, leading to long computational times. Whilst this problem can be parallelised, the number of pathways that contribute to a given measurement are extremely high (Supplementary Information Fig. S6), limiting the space of investigation. Here I address this by limiting the investigation to pathways that experience up to four TRs in the transverse plane. This accounts for ~ 99% of the measured DW-SSFP signal for the parameters investigated here (Fig. S6). However, this approximation would lead to amplified errors for investigations of tissue with long T_2_ or acquisitions with short TRs (Supplementary Information Fig. S4), and limits the framework to estimating and visualising the DW-SSFP signal, rather than its utility for parametric fits (model inversion). An approach to identify the most meaningful pathways that contribute to a measurement would facilitate extending the time-*dependent* framework to higher order pathways, but no robust method to achieve this was identified as part of this work. A key challenge is that the signal contributed from pathways that experience several TRs in the transverse plane is the average of many pathways with positive and negative signal amplitudes (Supplementary Information Fig. S9).

An alternative approach to investigate time-*dependent* systems is to utilise Monte Carlo simulations, which can be used to investigate the integration of sophisticated microstructural systems with the DW-SSFP sequence (Fig. 7b). Taken together, Monte Carlo simulations may provide the most generalisable approach to characterise time-*dependent* systems with DW-SSFP across a large parameter space, despite the long simulation times required to approach steady-state (typically requiring 10s to 100s of TRs—Supplementary Information Fig. S10). The proposed time-*dependent* pathway framework would provide complementary information to visualise how the estimated diffusion attenuation relates to the diffusion spectrum, providing both understanding and intuition of the signal-forming mechanisms of DW-SSFP.

### Comparisons with DW-SE and DW-STE

The description of DW-SSFP as a weighted sum of b-values/$\tilde{q}\left( \omega \right)$ profiles is a mathematically exact representation for time-*independent* and -*dependent* (under the GPA) systems. The equivalent mathematical representations for the DW-SE and DW-STE sequence corresponds to a single signal-forming magnetisation pathway associated with a single b-value (orange circles, Fig. 5) or $\tilde{q}\left( \omega \right)$ profile (Figs. 6b and 7a). Notably, the DW-SE sequence has been previously integrated into an EPG framework, deriving a signal amplitude and b-value consistent with DW-SE literature^36^.

This provides an approach to perform comparisons of microstructural estimates derived from DW-SSFP and DW-SE/STE data. Specifically, we can define a single biophysical model and perform comparisons of the microstructural estimates derived from a single b-value or weighted-sum of multiple b-values (alternatively, a single $\tilde{q}\left( \omega \right)$ profile versus multiple $\tilde{q}\left( \omega \right)$ profiles). Data acquired from DW-SSFP/SE/STE sequences could be similarly combined for simultaneous estimation of microstructural parameters. For example, for a time-*independent* system, this could be performed via simultaneous modelling the DW-SE/STE data with a single b-value and the DW-SSFP data with a weighted sum of b-values (e.g. Eqs. (10) and (12)). For a time-*dependent* system, this could be performed via simultaneous modelling of a single $\tilde{q}\left( \omega \right)$ and multiple $\tilde{q}\left( \omega \right)$) profiles via the GPA (Eq. (14)) to estimate a single $\tilde{D}\left( \omega \right)$ profile.

Comparisons between DW-SSFP and DW-SE sequence in this manuscript are defined in the context of an equivalent number of measurements and identical gradient waveforms and timings. Whilst this provides an insight into the conceptual differences in diffusion attenuation between the DW-SSFP and DW-SE sequence, it does not translate into a representation of the relative sensitivity of these sequences to a specific microstructural feature. Understanding this potential warrants further investigation to perform quantitative comparisons of the sensitivity of DW-SSFP and DW-SE sequences individually optimised for microstructural sensitivity and SNR, and is the subject of ongoing work^56^.

### Pathway thresholding

A key termination criterion in the implementation of the time-*independent* and -*dependent* frameworks is the number of TRs a given magnetisation pathway experiences in the transverse plane. This criterion was chosen as the signal contribution for a given number of TRs in the transverse plane arises from the cancellation of trillions/quadrillions/quintillions of magnetisation pathways with positive and negative signal amplitudes (Supporting Information Fig. S9). Defining termination criterions based on definitions of magnetisation pathway signal amplitudes can lead to incorrect estimates if these thresholds are too high, leading to unequal cancelation of positive and negative signals that contribute to the measured signal (Supporting Information Fig. S11). Termination criteria based on the number of TRs a magnetisation pathway experiences in the transverse plane also form the basis of existing partition framework representations of DW-SSFP^11,25^.

Given the high-dimensional parameter space of DW-SSFP (Fig. 2), the number of transverse TRs required to accurately characterise the signal depends on the specific DW-SSFP sequence parameters and sample properties. To support this evaluation, I provide software to estimate the accuracy of the non-diffusion weighted DW-SSFP signal estimate as a function of the number of transverse TRs. Evaluation as a function of the non-diffusion weighted DW-SSFP signal facilitates the optimisation of evaluations regimes where diffusion properties are unknown. It provides a lower bound for the accuracy of the transverse approximation, where it can be assumed that incorporation of diffusion effects will further improve accuracy (arising from increased attenuation of magnetisation pathways associated with additional TRs in the transverse plane). Supporting Information Fig. S12 provides some example characterisations of the accuracy as a function of the number of transverse TRs for different parameter sets, with the implementation software available at github.com/BenjaminTendler/SteadyStateDiffusionMicrostructure (AnalyticalModels/DWSSFP_TransversePeriodAccuracy.m).

### Generalisability of proposed framework

The proposed framework takes advantage of properties of spoiled, periodic sequences to efficiently incorporate the effects of time-*independent* and -*dependent* diffusion phenomena. Whilst this manuscript has been framed in the context of DW-SSFP, signal-forming echoes associated with alternative spoiled, periodic sequences (e.g. DESS, TESS) could be integrated and explored using the same methodology. Extensions of the framework to incorporate balanced and non-periodic sequences has not been investigated.

## Conclusion

I established a framework providing forward predictions of the measured DW-SSFP signal under a broad range of existing biophysical models incorporating time-*independent* and -*dependent* diffusion phenomena. The framework facilitates the visualisation and characterisation of the measured DW-SSFP signal, providing both understanding and intuition of how sequence properties influence diffusion attenuation. When considering time-*independent* diffusion, the framework additionally provides an approach to integrate and perform parameter estimation from experimental data, without the requirement of model-specific derivations. Findings give excellent agreement with existing analytical and simulation methods, demonstrating that DW-SSFP achieves microstructural sensitivity by investigating multiple b-values/$\tilde{q}\left( \omega \right)$ profiles simultaneously. Combined with its theoretical SNR-efficiency and low-distortion properties, the DW-SSFP sequence may offer considerable potential for characterising tissue microstructure, particularly at ultra-high field.

## Methods

### Default parameters and evaluation software

Default sequence parameters for all simulations (unless explicitly stated) were matched to the experimental DW-SSFP analysis at 7T performed in Tendler et al.^32^, setting ${\text{G}} = 52\;{\text{mT}}/{\text{m}}$, ${\updelta } = 13.56\;{\text{ms}}$, ${\upalpha } = 24^{{\text{o}}}$ and TR $= 28\;{\text{ms}}$, with ${\text{D}} = 0.2\;{\upmu \text{m}}^{2} /{\text{ms}}$, ${\text{T}}_{1} = 600\;{\text{ms}}$ and ${\text{T}}_{2} = 40\;{\text{ms}}$. Investigations were performed using MATLAB (2023a, The MathWorks, Inc., Natick, MA) on a Macbook Pro (macOS Big Sur, M1 chip, 16GB ram).

For the oscillating gradient investigations, parameters were matched to Aggarwal et al.^45^, setting $G = 674 \;{\text{mT}}/{\text{m}}$, $\delta = 20\; {\text{ms }}$ and ${\text{TR}}/{\Delta } = 25\; {\text{ms}}$. All other parameters were matched to those defined in the previous paragraph.

### Time-*independent* framework

Details of the time-*independent* framework are provided in the “Theory” section. For the practical implementation, a pathway was characterised as contributing to the measured signal if it had an amplitude (fraction of M_0_) above a defined threshold, here set to 10^−28^. I excluded pathways that persisted for over 1000 TRs (maximum relaxation attenuation ${\text{O}}(10^{ - 24} )$) and dictionary entries persisting for more than 10 TRs in the transverse plane (maximum relaxation attenuation ${\text{O}}(10^{ - 4} )$). These thresholds were found to have a negligible impact on signal estimation in the investigated regimes. Gradient waveforms were discretised into 1000 time points per TR.

### Time-dependent framework

Details of the time-*dependent* framework are provided in the “Theory” section. Eq. (14) was implemented using the approach described in Ning et al.^30^, reparameterising the GPA as a function of the mean-squared displacement of the diffusion system and the autocorrelation function of the gradient waveform. This approach was chosen as it can more rapidly estimate the signal attenuation when compared to Eq. (14). An analytical form of the mean squared displacement of a cylinder was defined based on Equation 32 in Mortensen et al.^57^.

I characterised a pathway as contributing to the measured signal if it had an amplitude (fraction of M_0_) above a defined threshold, here set to 10^−10^. I excluded pathways that persisted for over 1000 TRs (maximum relaxation attenuation ${\text{O}}(10^{ - 24} )$) or more than 4 TRs (maximum relaxation attenuation ${\text{O}}(10^{ - 2} )$) in the transverse plane, corresponding to ~ 99% of the measured signal for the investigated parameters (Supplementary Information Fig. S6). Gradient waveforms were discretised into 100 time points per TR. The average time to estimate a single data point for the cylindrical restriction investigation (Fig. 8c,d) was approximately 70 s.

### Monte Carlo simulations

Spin trajectories for all Monte Carlo simulations were generated using the Camino *datasynth* function^58^, modified to produce trajectories that followed a Gaussian distribution of displacements per time-step. The DW-SSFP and DW-SE signal was subsequently modelled using custom MATLAB code (2023a, The MathWorks, Inc., Natick, MA). All simulations used $5 \cdot 10^{5}$ spins.

For DW-SSFP, I simulated 10,000 time-steps (100 time-steps per TR, 100 TRs). These parameters ensured the measured signal reached a steady state (Supplementary Information Fig. S10). for DW-SE, I simulated 200 time-steps (200 time-steps per TR, 1 TR). For the DW-SSFP simulations, the initial position of spins was modified to match the $2\pi$ dephasing condition of DW-SSFP.

### Experimental DW-SSFP acquisition and analysis

Acquisition of the experimental data used in this manuscript is described in Tendler et al.^32^. Briefly, DW-SSFP data of a whole, human post-mortem brain was acquired with the following parameters: resolution $= { }850\;{{ \upmu m }}$ iso., no. directions $= { }120$, ${\text{q }} = 300\;{\text{ cm}}^{ - 1}$, ${\text{G }} = { }52{ }\;{\text{mT}}/{\text{m}}$, ${\delta } = { }13.56{ }\;{\text{ms}}$, ${\alpha } = { }24^{{\text{o}}} { }$ and $94^{{\text{o}}}$, ${\text{TE }} = { }21{ }\;{\text{ms}}$, ${\text{TR }} = { }28\;{\text{ ms}}$, Bandwidth $= { }393{ }\;{\text{Hz}}/{\text{mm}}$, Acquisition time per direction $= { }5\;{\text{m }}\;47\;{\text{s}}$, acquisition time per flip angle $= \sim { }12\;{\text{h}}$. Voxelwise quantitative T_1_, T_2_ and B_1_ maps were additionally derived using complementary acquisitions due to DW-SSFP’s dependence on relaxation times and flip angle (Fig. 2). The original processed data associated with this project can be accessed via the Digital Brain Bank^59^ (*Human ALS MRI-Histology dataset*).

For fitting to experimental data, I used the time-*independent* framework to generate a b-value distribution dictionary for a range of T_1_ (100 to 1000 ms; 10 ms increments), T_2_ (1 to 60 ms; 1 ms increments), and flip angles (1° to 140°; 1° increments). The final dictionary contained 764,400 b-value distributions, requiring ~ 20 GB of space. This dictionary was generated over several days on a Macbook Pro (macOS Big Sur, M1 chip, 16GB ram). However, this process can be readily parallelised and only need to be performed once for a given set of DW-SSFP parameters.

Fitting for the time-*independent* pathway framework was performed using the b-value distribution dictionary and Eq. (10) integrating the tensor representation (Eq. (12)). The comparison analytical DW-SSFP model^33^ with integrated tensor is described in Appendix 3. Fitting was performed with MATLAB (2023a, The MathWorks, Inc., Natick, MA) using *lsqnonlin*, processed on the computing cluster based at the Wellcome Centre for Integrative Neuroimaging.

For the experimental NODDI investigation, I similarly integrated the b-value distribution dictionary with Eq. (10) incorporating NODDI^43^ (Eq. (13)). Parameter estimation was performed via a multi-step pipeline, reflecting the implementation steps performed by the NODDI toolbox. Briefly, the principal fibre orientation ($\vec{V}_{1}$) was first initialised based on a tensor $\vec{V}_{1}$ estimate. The isotropic volume fraction ($f_{iso}$), intracellular volume fraction ($f_{intra}$) and orientation dispersion ($OD$) were subsequently estimated based on a custom grid-search fitting algorithm. To account for fixed post-mortem tissue, the intrinsic free diffusion coefficient ($D_{|| }$) was fixed to 0.42 ${\upmu \text{m}}^{2} /{\text{ms}}$, estimated based on a custom implementation of the spherical mean technique^60^ for DW-SSFP data. The isotropic diffusion coefficient ($D_{iso}$) was fixed to $2 {\upmu \text{m}}^{2} /{\text{ms}}$. The remaining free parameters ($f_{iso} , \;f_{intra} ,\; OD$ and $\vec{V}_{1}$) were simultaneously estimated using MATLAB *lsqnonlin*. Fitting was performed using a combination of custom MATLAB code and functions from the NODDI toolbox. It was not possible to investigate the existence of a dot compartment based on the acquired DW-SSFP data, with incorporation leading to noisy parameter estimates.

### Ethics statement

Experimental data derived from a whole, human post-mortem brain (Fig. 9) used tissue provided by the Oxford Brain Bank, a research ethics committee (REC) approved, HTA regulated research tissue bank. The study was conducted under the Oxford Brain Bank’s generic Research Ethics Committee approval (15/SC/0639). Informed consent was obtained by the Oxford Brain Bank. The research was performed in accordance with relevant guidelines and regulations.

### SNR-efficiency comparison

SNR-efficiency is defined as:

15 $${\text{SNR}}_{{{\text{eff}}}} = S \cdot \surd \rho ,$$

where $S$ is the estimated signal amplitude from the DW-SSFP, DW-SE and DW-STE sequence (Appendix 4), and $\rho = \sqrt {\frac{{{\text{T}}_{{{\text{acq}}}} }}{{{\text{TR}}}}}$, where ${\text{T}}_{{{\text{acq}}}}$ is the readout duration of each sequence (Appendix 5). Estimation of parameters that maximised ${\text{SNR}}_{{{\text{eff}}}}$ at a target b-value was performed using the *lsqnonlin* function in MATLAB (2023a, The MathWorks, Inc., Natick, MA), assuming free Gaussian diffusion. For conventional DW-SSFP (Fig. 10a–c), optimisation was performed for a target b-value by optimising:

16 $$\max \left\| {{\text{SNR}}_{{{\text{eff}}}} } \right\|_{2}^{2} + \frac{1}{{b_{{{\text{target}}}} }}\left\| {b_{{{\text{target}}}} - b_{{{\text{eff}}}} } \right\|_{2}^{2} ,$$

where $b_{{{\text{target}}}}$ was fixed to $10 {\text{ms}}/{\upmu \text{m}}^{2}$ and $b_{{{\text{eff}}}}$ is an effective DW-SSFP b-value that achieves the same level of diffusion attenuation as a conventional DW-SE or DW-STE sequence:

17 $$b_{{{\text{eff}}}} = - \frac{{\ln S/S_{0} }}{D}.$$

Optimisations were performed assuming no dead time, readout duration limits, gradient duty cycle limits or T_2_^’^ decay. These properties are expected to vary depending on the precise implementation of the imaging sequence and properties of the MR system.

For the optimisation, T_1_, T_2_ and D were set equal to 552 ms, 26.8 ms and 0.14 μm^2^/ms, the median values estimated in white matter at 7T from a cohort of post-mortem brains fixed with neutral buffered formalin^61^. This cohort included the brain used for the Tensor and NODDI investigation (Fig. 9).

## Supplementary Information

Supplementary Information 1.

Supplementary Information 2.

## Data Availability

Software for the time-*independent* and -*dependent* frameworks, including scripts to replicate many of the findings presented in this manuscript, are available at github.com/BenjaminTendler/SteadyStateDiffusionMicrostructure. The original processed post-mortem data associated with this project can be accessed via the Digital Brain Bank^59^ (*Human ALS MRI-Histology dataset*).
