# Supplementary material for: Investigating time-independent and time-dependent diffusion phenomena using steady-state diffusion MRI
Source: Sci Rep. 2025 Jan 28;15:3580. doi: 10.1038/s41598-025-87377-x (PMC11775203; doi:10.1038/s41598-025-87377-x)
Supplement: Supplementary file 1 — Supplementary Information 1. [file 41598_2025_87377_MOESM1_ESM.pdf]

Appendices

Appendix 1

Based on the proposed analytical model in Freed et al.[1], the DW-SSFP sequence incorporating a diffusion gradient of duration δ is modelled as:

$$S_{\mathrm{DW-SSFP}} = -r_1 \cdot b_0, \qquad \text{[A1]}$$

where:

$$b_0 = \frac{-\sin\alpha \cdot S_0(1 - E_{1,0})}{A_{p=0} - B_{p=0} + E_{2,-1} \cdot C_{p=0} \cdot r_1} \qquad \text{[A2]}$$

$$r_1 = \frac{1}{E_{2,-1} \cdot B_{p=0}} x_1 + \frac{E_{2,0} \cdot C_{p=1}}{B_{p=1}} \qquad \text{[A3]}$$

defining:

$$A_p = \frac{1}{2} \cdot \left(E_{1,p} - 1\right) \cdot (1 + \cos\alpha) \qquad \text{[A4]}$$

$$B_p = \frac{1}{2} \cdot \left(E_{1,p} + 1\right) \cdot (1 - \cos\alpha) \qquad \text{[A5]}$$

$$C_p = E_{1,p} - \cos\alpha \qquad \text{[A6]}$$

$$E_{1,p} = e^{-\frac{\mathrm{TR}}{T_1} - D \cdot \gamma^2 \cdot G^2 \cdot \delta^2 \cdot p^2 \cdot \mathrm{TR}} \qquad \text{[A7]}$$

$$E_{2,p} = e^{-\frac{\mathrm{TR}}{T_2} - D \cdot \gamma^2 \cdot G^2 \cdot \delta^2 \cdot \left(\left(\mathrm{p}^2 + \mathrm{p} + \frac{1}{3}\right) \cdot \delta + (\mathrm{p}+1)^2 \cdot (\mathrm{TR} - \delta)\right)} \qquad \text{[A8]}$$

$$x_1 = \cfrac{n_1}{d_1 + \cfrac{n_2}{d_2 + \cfrac{n_3}{d_3 + \cdots + \cfrac{n_l}{d_l + e_l}}}}, \qquad \text{[A9]}$$

with:

$$n_p = -E_{2,-p} \cdot E_{2,p-1} \cdot A_p^2 \cdot \frac{B_{p-1}}{B_p} \qquad \text{[A10]}$$

$$d_p = \left(A_p - B_p\right) + E_{2,-p-1} \cdot E_{2,p} \cdot \frac{B_p}{B_{p+1}} \cdot C_{p+1} \qquad \text{[A11]}$$

$$e_p = -E_{2,-p-1} \cdot E_{2,p} \cdot \frac{B_p}{B_{p+1}} \cdot C_{p+1}. \qquad \text{[A12]}$$

Find the associated code for this model here.

Appendix 2

The DW-SSFP Analytical model for oscillating gradients is equivalent to Appendix 1, setting:

$$E_{1,p} = e^{-\frac{\mathrm{TR}}{T_1}} \quad \text{[A13]}$$

$$E_{2,p} = e^{-\frac{\mathrm{TR}}{T_2} - D \cdot b}, \quad \text{[A14]}$$

where b is equivalent to a b-value of a single instance of the oscillating gradient waveform (Figure 7a; here $b = 0.35\ \mathrm{ms/\mu m^2}$). Find the associated code for this model here.

Appendix 3

The DW-SSFP Analytical model with integrated tensor is equivalent to Appendix 1, setting:

$$D = \hat{g}_k^T \boldsymbol{D} \hat{g}_k, \quad \text{[A15]}$$

where $\hat{g}_k$ is the gradient orientation and $\boldsymbol{D}$ is the diffusion tensor. Find the associated code for this model here.

Appendix 4

$S_{\mathrm{DW-SSFP}}$ is defined in Appendix 1, with

$$S_{\mathrm{DW-SE}} = S_0 \cdot \left(1 - e^{-\frac{\mathrm{TR}}{T_1}}\right) \cdot e^{-\frac{\mathrm{TE}}{T_2}} \cdot e^{-b \cdot D}, \quad \text{[A16]}$$

$$S_{\mathrm{DW-STE}} = \frac{S_0}{2} \cdot \left(1 - e^{-\frac{\mathrm{TR} - \mathrm{T_{mix}}}{T_1}}\right) \cdot e^{-\frac{\mathrm{T_{mix}}}{T_2}} \cdot e^{-\frac{\mathrm{TE}}{T_2}} \cdot e^{-b \cdot D}, \quad \text{[A17]}$$

where $\mathrm{T_{mix}}$ is the mixing time.

Appendix 5

$$\rho_{\mathrm{DW-SSFP}} = \frac{\mathrm{TR} - \delta}{\mathrm{TR}}, \quad \text{[A18]}$$

$$\rho_{\mathrm{DW-SE}} = \frac{2 \cdot (\Delta - \delta)}{\mathrm{TR}}, \quad \text{[A19]}$$

where Δ is the diffusion time, defining $\mathrm{TE} = 2 \cdot \Delta$ (i.e. no dead time between the RF pulse and the start of the second diffusion gradient), and

$$\rho_{\mathrm{DW-STE}} = \frac{\mathrm{TE} - 2 \cdot \delta}{\mathrm{TR}}. \quad \text{[A20]}$$

References

1. Freed, D. E., Scheven, U. M., Zielinski, L. J., Sen, P. N. & Hürlimann, M. D. Steady-state free precession experiments and exact treatment of diffusion in a uniform gradient. *J. Chem. Phys.* (2001).
